# *Momordicae Semen* inhibits migration and induces apoptotic cell death by regulating c-Myc and *CNOT2* in human pancreatic cancer cells

**DOI:** 10.1038/s41598-023-39840-w

**Published:** 2023-08-07

**Authors:** Wona Jee, Hyun Min Ko, Do-Il Park, Ye-Rin Park, So-Mi Park, Hyungsuk Kim, Yun-Cheol Na, Ji Hoon Jung, Hyeung-Jin Jang

**Affiliations:** 1 College of Korean Medicine, Kyung Hee University, 26, Kyungheedae-Ro, Dongdaemun-Gu, Seoul, 02447 Korea; 2 Department of Science in Korean Medicine, Graduate School, Kyung Hee University, Seoul, 02447 Korea; 3 Department of Korean Rehabilitation Medicine, College of Korean Medicine, Kyung Hee University, Seoul, Korea; 4 Western Seoul Center, Korea Basic Science Institute, 150 Bugahyeon-Ro, Seodaemun-Gu, Seoul, 03759 Republic of Korea

**Keywords:** Cancer, Cell biology, Oncology

## Abstract

Pancreatic cancer(PC) is less common than other cancers; however, it has a poor prognosis. Therefore, studying novel target signaling and anticancer agents is necessary. Momordicae Semen (MS), the seed of Momordica sochinensis Spreng, mainly found in South-East Asia, including China and Bangladesh, is used to treat various diseases because of its anticancer, antioxidant, anti-inflammatory, and antibacterial properties. However, the effect of the MS extract on pancreatic cancer cells remains unknown. In this study investigated whether the MS extract exerted an anti-cancer effect by regulating c-Myc through CNOT2. Cytotoxicity and proliferation were investigated using MTT and colony formation assays. The levels of apoptotic, oncogenic, and migration-associated factors were confirmed using immunoblotting and immunofluorescence. Wound closure was analyzed using a wound healing assay. The chemical composition of the MS methanol extracts was analyzed using liquid chromatography–mass spectrometry. We confirmed that the MS extract regulated apoptotic factors and attenuated the stability of c-Myc and its sensitivity to fetal bovine serum. Furthermore, the MS extract increased apoptosis by regulating c-Myc and CNOT2 expression and enhanced the sensitivity of 5-FU in pancreatic cancer. This study showed that the MS extract is a promising new drug for PC.

## Introduction

In 2020, pancreatic cancer was the 7th most common cancer in men and women, accounting for 2.6 % of cases and 4.7 % of mortalities from all cancer-causing sites^1,2^. Additionally, pancreatic cancer is one of the cancers with the worst prognosis because it easily metastasizes to nearby organs or lymph nodes^3^. Recent studies reported that FOLFIRINOX therapy consists of gemcitabine, capecitabine or folinic acid, fluorouracil, irinotecan, and oxaliplatin is the first-line adjuvant chemotherapy for pancreatic cancer^4^. Although many studies have been conducted on pancreatic cancer, the response to chemotherapy is limited, and the 5-year survival rate of patients with PC has not improved over the past 30 years. Furthermore, pancreatic cancer is difficult to detect in its early stages and metastasizes quickly; making it challenging to treat through surgery^5^. Therefore, there is a need to identify novel biomarkers and anticancer agents with clear relevance to pancreatic cancer^6^.

The c-Myc oncogene has been implicated in the pathogenesis of several cancers, including pancreatic, hepatocellular, and breast cancers. c-Myc is a transcription factor that regulates cell life and death, including metabolism, mitochondrial function, ribosome biosynthesis, differentiation, apoptosis, and cancer cell proliferation^7–9^. In humans, c-Myc, located on chromosome 8, binds to the enhancer box (E-box) to regulate gene expression. Like many other transcription factors, MYC is an unstable protein degraded by ubiquitination, such as the Ub ligase Skp2, which regulates c-Myc stability. c-Myc is also overexpressed in primary pancreatic cancer at a rate of 43.5%. Overexpression of c-Myc induces the conversion of pancreatic intraepithelial neoplasm (PanIN) to pancreatic cancer, and c-Myc inhibition suppresses cell proliferation in PC in vitro and induces chemotherapy sensitivity in vivo^10–12^. Furthermore, c-Myc signaling mediated by CNOT2 and the ribosomal proteins L5 or L11 regulate cell growth in hepatic cancer cells^13^.

CNOT2 is one of the nine subunits of the CCR4-NOT complex (CNOT) that regulates transcription and translation. Additionally, CNOT2 is an oncogene that promotes lipid metabolism, angiogenesis, proliferation, and autophagy^13,14^. A recent study reported that inhibition of CNOT2 in human cancer cells inhibits cancer cell proliferation and angiogenesis through vascular endothelial growth factor (VEGF) signaling in cancer cells, suggesting that CNOT2 acts as an oncogene^15^. However, the role of CNOT2 in PC has not been elucidated.

Herbal medicines have good therapeutic effects with few side effects; therefore, researchers have focused on the effects of natural products^16^. The herbal medicine used in this study, *Momordica Semen* (MS), is the seed of *Momordica sochinchinensis Spreng*, which belongs to the *Cucurbitaceae* family and is mainly distributed in Southeast Asian countries, such as China and Bangladesh^17^. MS has been used therapeutically for a long time in oriental medicine to alleviate various symptoms such as hemangiomas, furuncles, cramps, tooth decay, and polymyalgia rheumatica. MS has been used in treating various diseases owing to its anticancer (breast, melanoma, lung, gastric, and cervical cancer), antioxidant, anti-inflammatory, antibacterial, and renal cell protective properties^18^. However, the effect of the MS extract on pancreatic cancer cells remains unknown.

In this study, we evaluated the anticancer effects of the MS extract through the regulation of c-Myc and CNOT2 in pancreatic cancers. In addition, we performed a chemical composition analysis of the MS methanol extract using liquid chromatography–mass spectrometry (LC-MS).

## Results

### MS extract inhibits cell proliferation in pancreatic cancer cells

**Figure 1:**
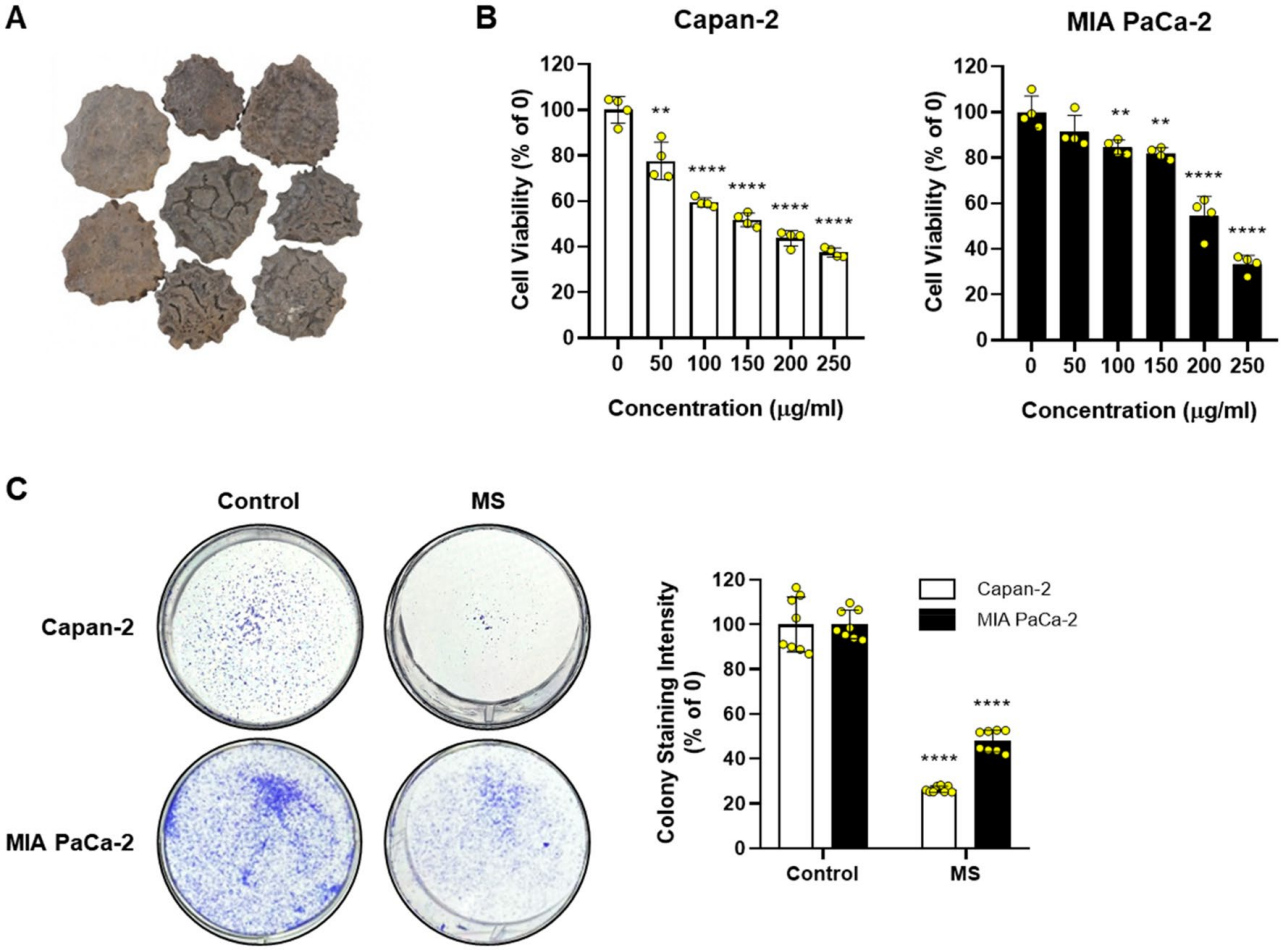
Inhibitory effect on cell proliferation in pancreatic cancer cells. (**A**) Seeds of *Momordica cochinchinensis* Sprenger (Momordicae Semen, MS). The image of the medicinal material used in this study was obtained from the Korean Medicine Resources Research Center, Korea Institute of Oriental Medicine. (**B**) Cells were seeded in 96-well plates and treated with MS extract or DMSO for 24 h. Cell viability was determined using the MTT assay. (**C**) Cells treated with or without MS were reseeded in 6-well plates at 1 × 10^3^ and incubated for 14 days. Colony formation was stained with a Diff Quik kit, and the data were determined by measuring absorbance after dissolving in ethanol. The data were quantified based on only DMSO treated group of each cell. *P* < 0.01, *P* < 0.001 and *P* < 0.0001 compared to the condition with zero treatment concentration.

The MTT assay was conducted to elucidate the cytotoxicity of MS in Capan-2 and MIA PaCa-2 pancreatic cancer cells. We confirmed that the cell viability declined significantly with increasing MS concentrations. Accordingly, MS showed powerful inhibition of Capan-2 and MIA PaCa-2 cell viability in a dose-dependent manner (Fig. 1B). We performed a colony formation assay to determine the effect of long-term treatment with MS. Consistently, MS (50–200 μg/mL) hindered colony formation more effectively than the control (Fig. 1C). These data indicated that MS effectively interrupts pancreatic cancer cell proliferation and induces apoptosis.

### LC–MS chromatography of MS methanol extract

**Figure 2:**
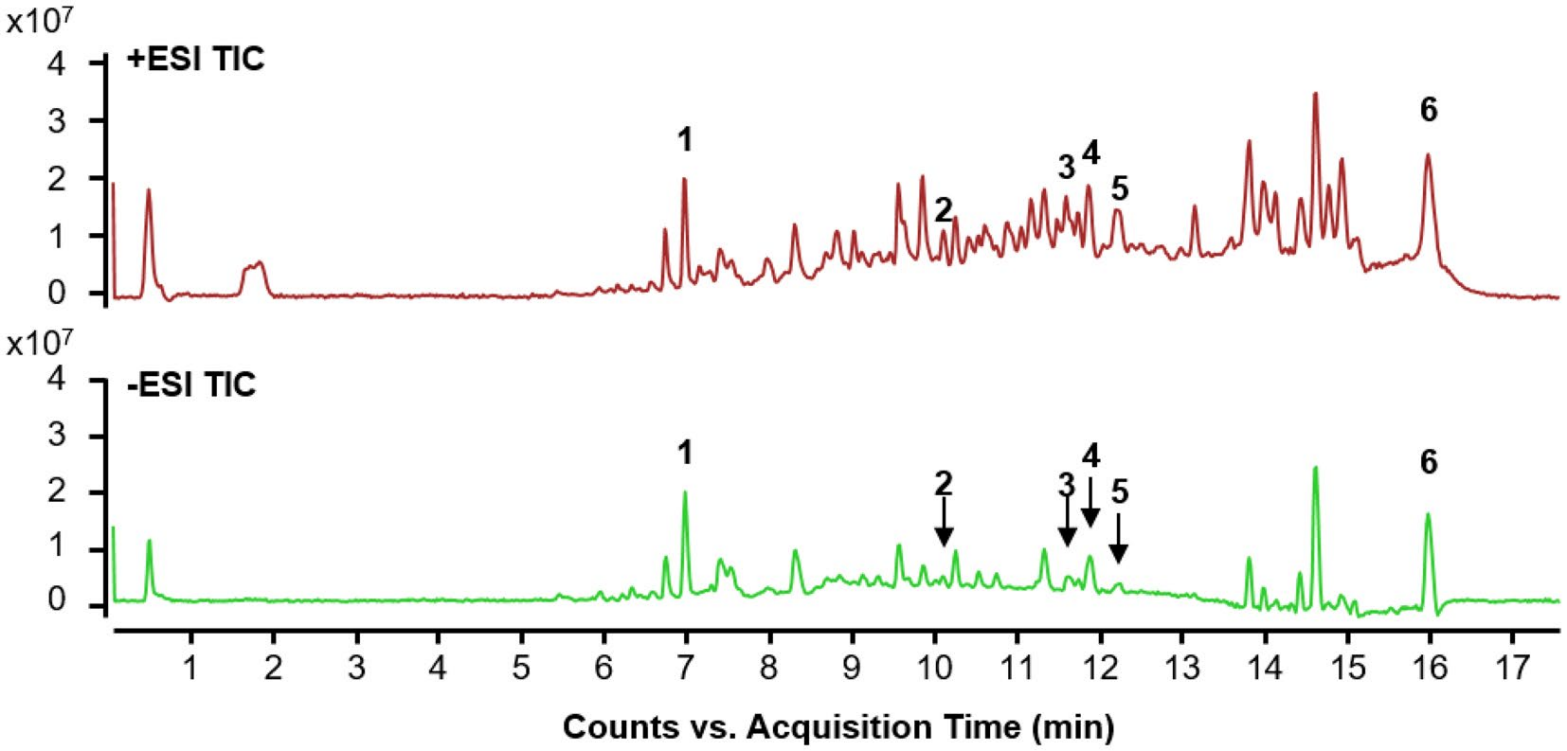
LC–MS chromatography of MS methanol extract. All chromatograms were monitored at ESI + and ESI-. The y-axis represents the intensity of the peak and the x-axis represents the retention time of the peak. The names of compounds corresponding to each peak number are as follows; 1: Kaempferol, 2: Apigenin, 3: Chlorogenic acid, 4: Hexadec-1 enoic acid, 5: Myricetin, 6: Saponin II.

We performed LC-MS analysis to identify the major components of the anticancer effect of pancreatic cancer cells. Most of the possible adducts were recognized in ES+ and ES- cells as follows and were used to analyze the LC-MS data qualitatively: ESI+: [M + H]+, [M + Na]+, and [M + K]+; ESI-: [M + H2O]-, [M + Br]-, and [M + COOH]-. The LC-MS chromatogram of the MS extract showed six peaks, indicating the presence of six bioactive components (Fig. 2). Table 1 shows the compounds identified by analyzing their mass spectra.

**Table 1:** List of compounds detected in MS Methanol extracts using LC–MS.

No	Compound	R.T (min)	Mass	Molecular Formula	Experimental Mass (m/z)	Selected Ion species
1	Kaempferol	6.974	286.0477	C15H10O6	(+) 309.0442	(M + Na) +
					(−) 304.9132	(M + H2O)-
2	Apigenin	10.084	270.0528	C15H10O5	(+) 293.1972	(M + Na) +
					(−) 315.2497	(M + Br)-
3	Chlorogenic acid	11.59	354.0951	C16H18O9	(+)393.2488	(M + K) +
					(−) 433.2167	(M + Br)-
4	Hexadec-11-enoic acid	11.855	254.2246	C16H30O2	(+) 277.2072	(M + Na) +
					(−) 299.2464	(M + COOH)-
5	Myricetin	12.235	318.037	C15H10O8	(+) 319.2171	(M + H) +
					(−) 299.2464	(M + COOH)-
6	Saponin II	16.024	634.4081	C36H58O9	(+) 673.3712	(M + K) +
					(−) 713.3270	(M + Br)-

### MS extract induces apoptotic cell death in pancreatic cancer cells

**Figure 3:**
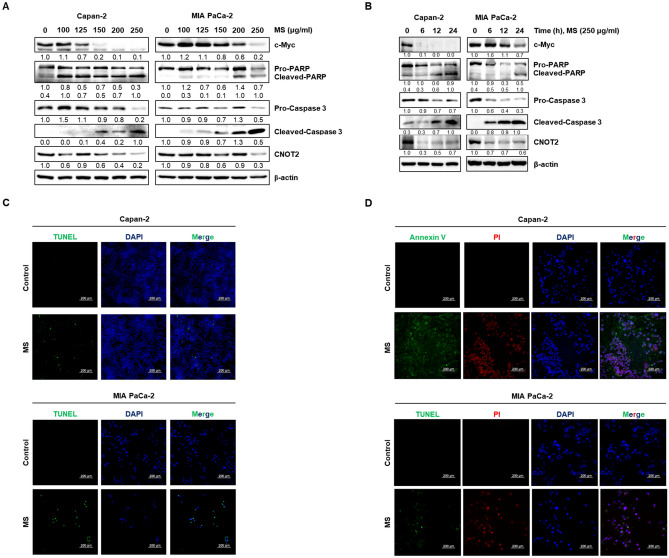
Apoptotic effect of MS extract in pancreatic cancer cells. Cells were treated with MS extract (**A**) at various concentrations for 24 h and (**B**) 250 μg/mL at the indicated time points. The protein expression of cleaved PARP and Caspase 3, which are apoptosis markers, and c-Myc and CNOT2, which are oncogenic factor, was confirmed by immunoblotting. The data were quantified compared to only DMSO treated groups. Cells treated with or not MS extract (250 μg/ml) were stained with apoptotic cells through TUNEL assay (**C**) and Annexin V/PI staining (**D**), and the magnification is 100x.

In human cancers, c-Myc plays a vital role as an oncogene, and it is involved in the cancer cells proliferation, cell cycle, metabolism, and survival. Previously, we showed that inhibition of CNOT2 induces apoptosis by regulating c-Myc^14^. Immunoblotting was performed using pancreatic cancer cells to confirm whether the MS extract regulates c-Myc and CNOT2. Figure 3A,B show that the MS extract inhibited the expression of c-Myc and CNOT2 in a dose- and time-dependent manner. Furthermore, we confirmed that the MS extract induced apoptosis by examining changes in PARP and caspase3. We analyzed whether the decrease in cell viability caused by the MS extract resulted from apoptosis using the TUNEL assay and Annexin V/PI staining. The results showed that TUNEL-positive cells and Annexin V/PI-positive cells increased after treatment with the MS extract in MIA PaCa-2 cells and Capan-2 cells (Fig. 3C,D). These data suggested that the MS extract suppressed the protein expression of c-Myc and CNOT2 and induced apoptosis in pancreatic cancer cells.

### MS extract modulates c-Myc stability in pancreatic cancer cells

**Figure 4:**
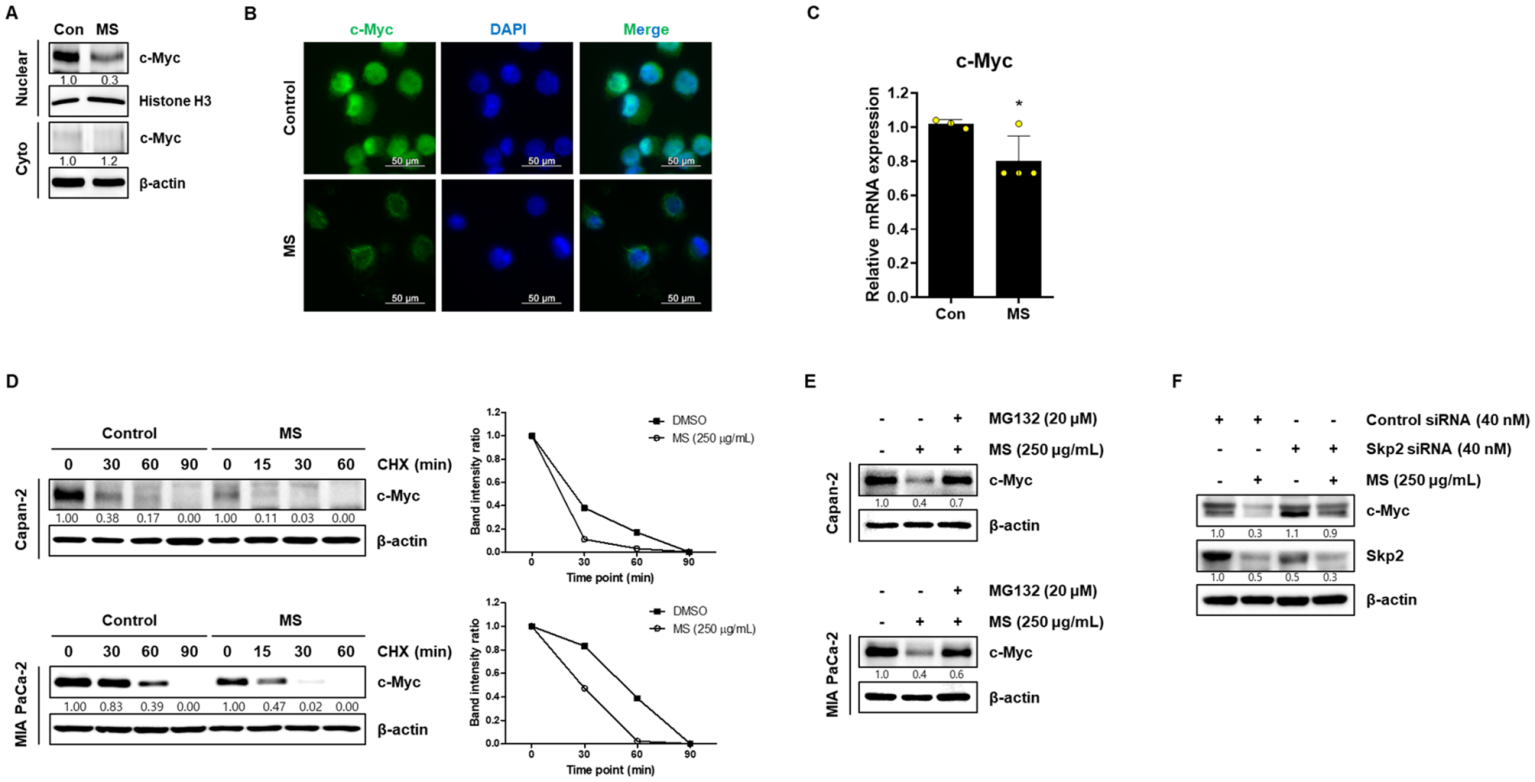
Modulation effects of c-Myc stability in pancreatic cancer cells. (**A**) The lysates were separated into nuclear and cytosolic fractions, and the protein expression of c-Myc was analyzed using immunoblotting. The data were normalized to a housekeeping gene in cell localization. (**B**) Cells were stained with c-Myc (green) and nucleus (blue) through immunofluorescence (magnification, × 400). (**C**) the relative mRNA level of c-Myc was detected using qRT-PCR with GAPDH as normalization. (**A**, **C**) The data were obtained using MIAPaCa-2, and quantified based on only the DMSO group. (**D**) Cells treated with DMSO or MS extract for 24 h were exposed to CHX in different time points to show the half-life of c-Myc protein. The data were normalized with β-actin, and quantified based on the condition of 0 min of CHX treatment in DMSO and MS groups. (**E**) MIA PaCa-2 transfected with Control siRNA or Skp2 siRNA for 48 h was treated with MS extract for 24 h, and protein expression levels of c-Myc and Skp2 were analyzed. The data were normalized with β-actin and quantified based on the first lane. *P* < 0.05 compared to the condition with zero treatment concentration.

We investigated the role of c-Myc in MS-induced apoptosis. Figure 4A and B show that c-Myc was mainly located in the nucleus and was significantly reduced by the MS extract in MIA PaCa-2 cells. The mRNA level of c-Myc was also significantly decreased by the MS extract (Fig. 4C). Furthermore, we confirmed the regulation of c-Myc protein expression by the MS extract. Moreover, compared to the Dimethyl sulfoxide (DMSO) (control) group, the MS extract reduced the stability of c-Myc half-life in Capan-2 and MIA PaCa-2 cells in the presence of cycloheximide (Fig. 4D). Skp2 is Known to regulated c-Myc stability by binding to its C-terminus and promoting its ubiquitination and subsequent degradation. Figure 4E shows that Skp2 knockdown increased c-Myc protein expression, while treatment with the MS extract reduced its expression, suggesting that the MS extract inhibits c-Myc protein stability.

### MS extract inhibits c-Myc sensitivity to FBS in pancreatic cancer cells

**Figure 5:**
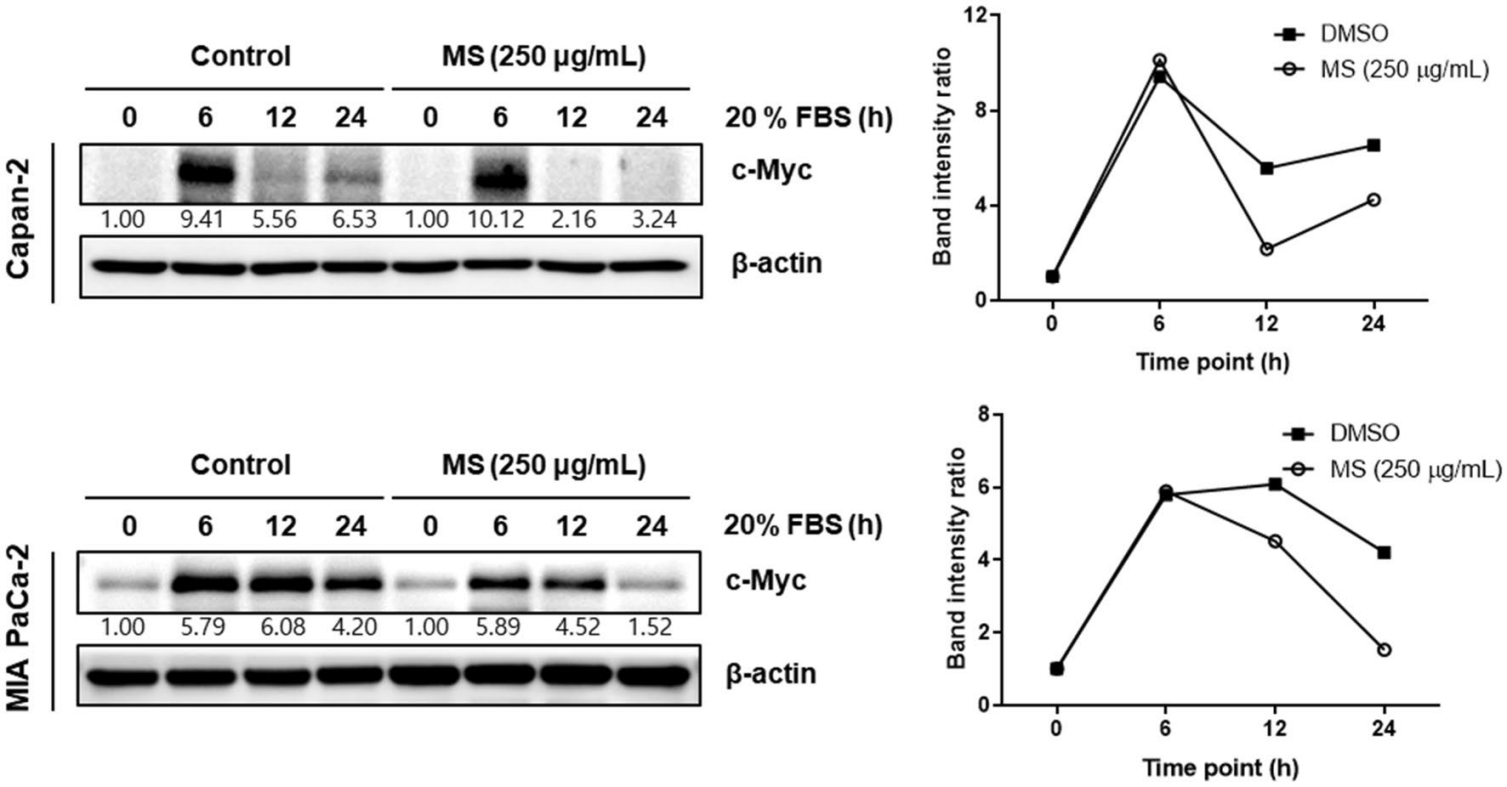
Inhibitory effect of c-Myc sensitivity to FBS in pancreatic cancer cells. After starvation for 16 h, 20% FBS containing MS extract (250 μg/mL) was treated at indicated time points. The protein expression of c-Myc was analyzed by immunoblotting. The data were normalized with β-actin and quantified based on the condition of 0 min of 20% FBS in DMSO and MS groups.

During serum stimulation, c-Myc responds rapidly. To confirm whether serum-responsive induction can be affected by the MS extract, we compared c-Myc expression in pancreatic cancer cells treated with the MS extract or DMSO by serum stimulation (Fig. 5). The expression of c-Myc is further decreased in the MS-treated group than in the DMSO-treated group 12 h after treatment, suggesting that MS regulates the expression of c-Myc after serum stimulation.

### CNOT2 is overexpressed in PC, and CNOT2 inhibition by MS extract induces apoptotic cell death by regulating c-Myc

**Figure 6:**
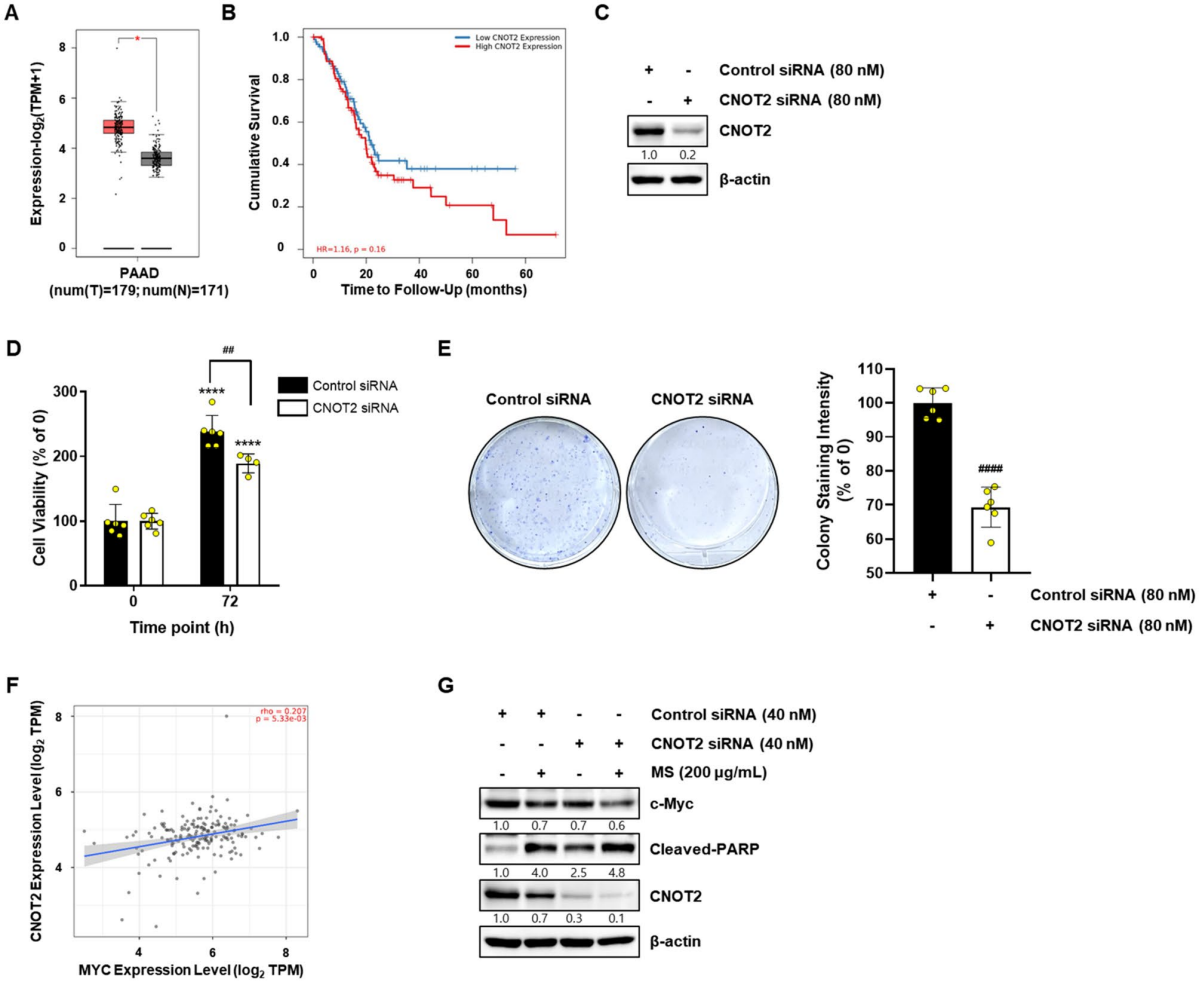
Expression pattern of CNOT2 in normal and tumor tissue, and the cell death effect by CNOT2 inhibition by MS extract treatment. (**A**) CNOT2 protein expression in Normal and tumor in pancreatic tissues. (**B**) The survival rate according to protein expression of CNOT2 in PAAD patients. MIA PaCa-2 cells were transfected with control siRNA or CNOT2 siRNA for 72 h. (**C**) The protein expression of CNOT2 was analyzed by immunoblotting. (**D**) Cells were seeded in 96-well plates and transfected with control siRNA or CNOT2 siRNA for 72 h. The cell viability was determined using the MTT assay. The data were quantified based on the condition of 0 h in control siRNA and CNOT2 siRNA groups. (**E**) Cells transfected with control siRNA or CNOT2 siRNA were reseeded in 6-well plates at 1 × 10^3^ and incubated for 14 days. Colony formation was stained with a Diff Quik kit, and the data were determined by measuring absorbance after dissolving in ethanol. (**F**) The relationship between CNOT2 and MYC in PAAD. (**G**) Cells were transfected with control siRNA or CNOT2 siRNA for 48 h, then treated with MS (200 μg/mL) for 24 h. The protein expression was analyzed by immunoblotting and normalized by β-actin. (**E**, **G**) The Data were quantified based on control siRNA treated group. *P* < 0.0001 compared to the within-group control condition and *P* < 0.01 and *P* < 0.0001 compared between group.

In a previous study, liver cancer growth was shown to regulate liver c-Myc mediated by CNOT2. We investigated the expression levels of the CNOT2 gene in adjacent normal and tumor tissues in pancreatic cancer using the Cancer Genome Atlas (TCGA) website. The results showed that CNOT2 was overexpressed more in tumor tissues than in normal tissues, indicating that CNOT2 regulates c-Myc expression in PC (Fig. 6A). TIMER2.0 is an oncogenomics analysis web tool based on TCGA data. Using this tool, a correlation analysis between CNOT2 gene expression pattern and pancreatic cancer patient survival rate was conducted, revealing that higher CNOT2 expression is associated with lower survival rate. These results suggest that the CNOT2 gene may play an important role in the development and progression of pancreatic cancer (Fig. 6B). Therefore, CNOT2 can be considered an important genetic marker in the treatment and prognosis evaluation of pancreatic cancer. CNOT2 knockdown in MIA PaCa-2 cells significantly inhibits cell survival and proliferation (Fig. 6C–E). The association between the expression of CNOT2 and MYC and the characteristics of pancreatic tumors was confirmed using TCGA analysis. We confirmed that CNOT2 and MYC had a significant proportional relationship with PC (Fig. 6F). Subsequently, we confirmed whether CNOT2 plays an essential role in c-Myc regulation via MS extract using siRNA. In Fig. 6G, CNOT2 deficiency by siRNA enhanced the inhibitory effect of MS on c-Myc expression compared to the control group. These results suggest that MS suppresses c-Myc expression in pancreatic cancer through CNOT2.

### MS extract inhibits cell migration by suppressing *CNOT2* in pancreatic cancer cells

**Figure 7:**
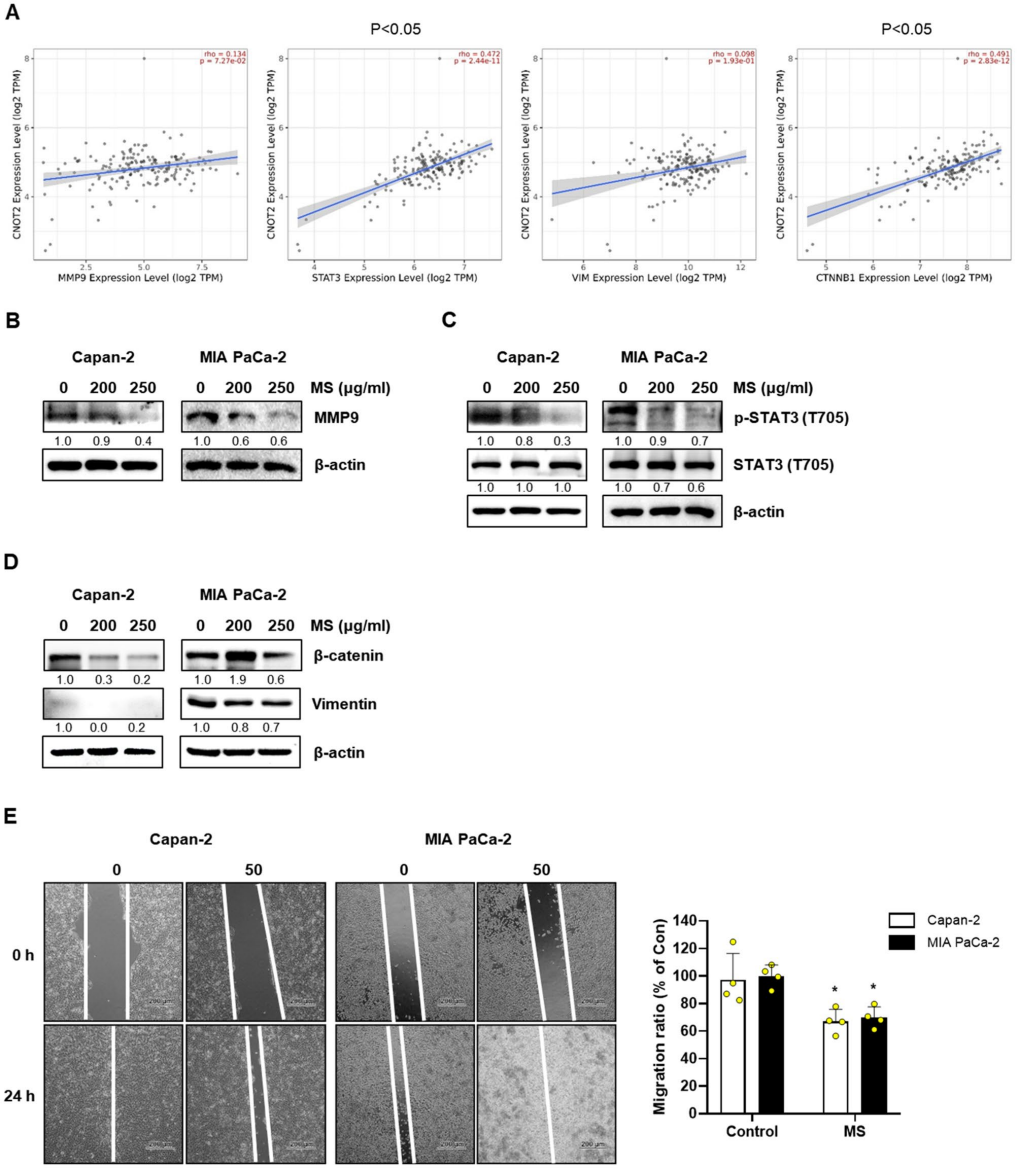
Inhibitory effect of cell migration by suppressing CNOT2 in pancreatic cancer cells. (**A**) Relationship between CNOT2 gene and migration associated gene in PAAD. (**B**–**D**) Pancreatic cancer cells were treated with MS extract (0, 200, 250 μg/mL) for 24 h. The protein expression of (**B**) MMP9, (**C**) p-STAT3, and (**D**) β–catenin, Vimentin is determined by immunoblotting. (**E**) The data were wound healing closure in pancreatic cancer cells. The data were normalized by β-actin and quantified based on 0 μg/mL treated group. *P* < 0.05 compared to the condition with zero treatment concentration.

A previous study reported that CNOT2 deficiency in breast cancer inhibited cell proliferation and angiogenesis via VEGF signaling. Figure 6 shows CNOT2 deficiency-induced apoptosis in pancreatic cancer cells. To investigate the association between CNOT2 and migration in PC, we analyzed the Cancer Genome Atlas (TCGA). As a result, we that STAT3 and CTNNB1 expression was significantly proportional to CNOT2 expression. MMP9 and VIM showed a proportional relationship with CNOT2; however, the results were insignificant (Fig. 7A). The protein expression level encoded by this gene was investigated by immunoblotting. When pancreatic cancer cells were treated with the MS extract, the expression of MMP9, β-catenin, and vimentin decreased in a concentration-dependent manner, and the phosphorylation of STAT3 also decreased. Additionally, the wound healing assay confirmed that the MS extract weakened wound closure in pancreatic cancer cells.

### MS extract showed combinational anticancer effect with 5-FU in pancreatic cancer cells

**Figure 8:**
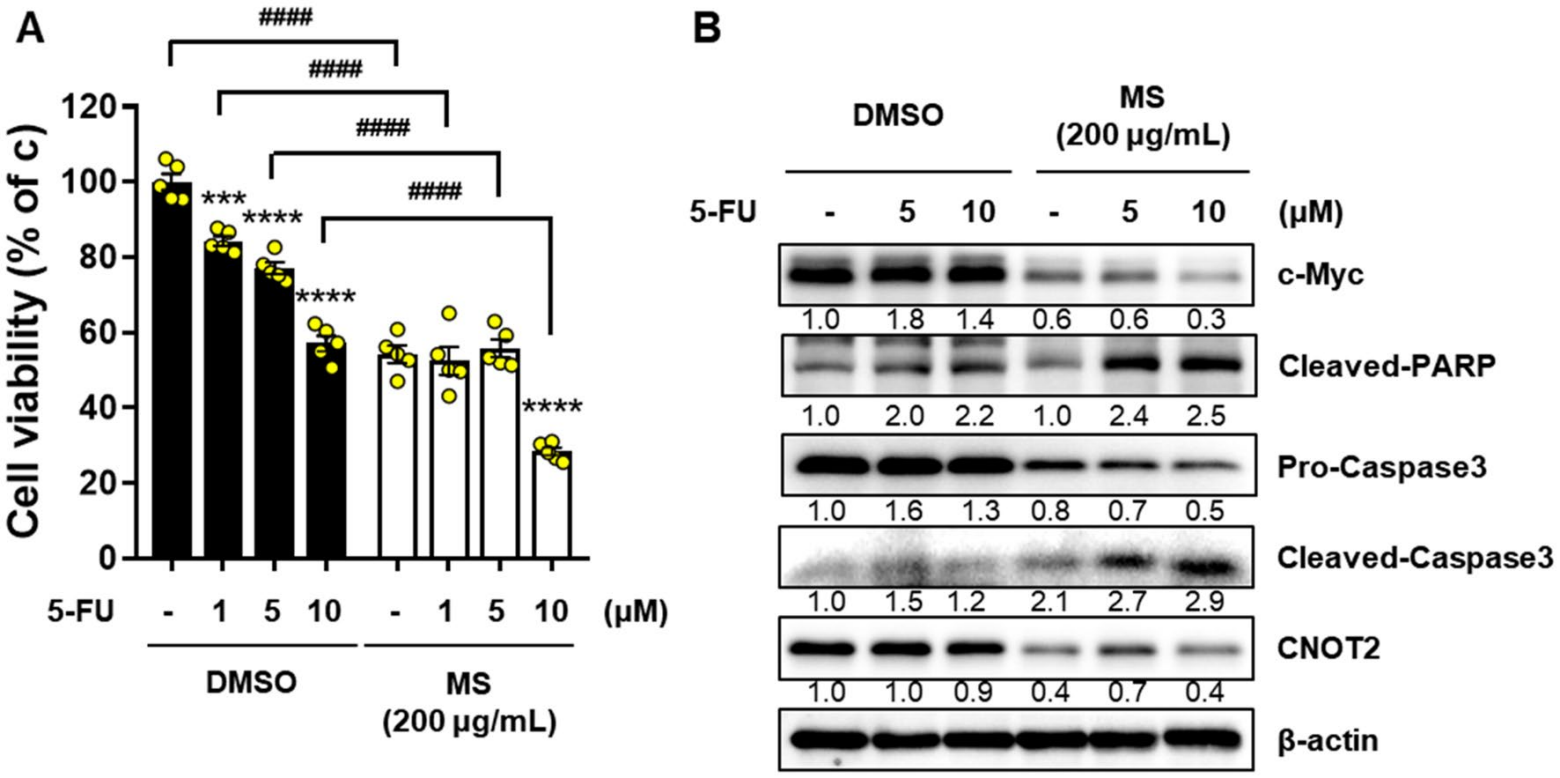
Combinational effect of MS extract and 5-FU in pancreatic cancer cells. 5-FU was treated with or not MS extract (200 μg/mL) for 24 h in MIA PaCa-2 cells. (**A**) Cell viability was determined by MTT assay, and (**B**) the protein expression of cleaved PARP and Caspase 3, which are apoptosis markers, and c-Myc and CNOT2, which are oncogenic factor, was confirmed by immunoblotting. The data were quantified compared to only DMSO-treated groups. *P* < 0.001 and *P* < 0.0001 compared to the within-group control condition and ^**####**^*P* < 0.0001 compared between groups.

The side effects of anticancer drugs during chemotherapy are difficult to overcome. To investigate the combined effect of 5-FU and the MS extract, 5-FU was treated with DMSO (control) or the MS extract in MIA PaCa-2 cells. Cell viability was significantly decreased in a concentration-dependent manner compared to the control group (Fig. 8A). The protein expression levels of the oncogenes c-Myc and CNOT2 and the apoptotic factors cleaved PARP and caspase 3 were decreased compared to the control group (Fig. 8B). Thus, we confirmed that the MS extract boosts the anticancer effect of 5-FU in pancreatic cancer cells.

## Discussion

The *KRAS* gene is frequently mutated at codon 12 in PC. It has the following characteristics: drug resistance, avoidance of apoptosis, reduced sensitivity to signals, infinite replication potential, and induction of angiogenesis, invasion, and metastasis^19^. Therefore, despite an abundance of various treatment methods for PC, the overall survival (OS) of patients has not improved. Therefore, there is a need to develop novel therapies and more efficacious therapeutic strategies that target defective signaling molecules and tumor microenvironments^20,21^. Despite active drug development and research on treating pancreatic cancer, the side effects of existing drugs and imperceptible responses to chemotherapy are difficult^22,23^. Therefore, it is important to identify novel therapeutics and targeted mechanisms for effective treatment. To solve this problem, this study demonstrated that MS extract is an efficacious treatment option for pancreatic cancer. To our knowledge, this is the first study to show that the MS extract induces cell death, including apoptosis, inhibition of migration, and cell proliferation, through c-Myc and CNOT2 in pancreatic cancer cells. Because peak identification is a basic level of compound discovery, it is necessary to identify effective compounds to evaluate the effectiveness of natural products. LC-MS analysis identified six components that could affect PC. Kaempferol is an antioxidant compound present in fruits and vegetables. It mainly promotes anti-inflammatory and antioxidant properties and has important neuroprotective effects in various diseases, such as cancer. In addition, it exerts a protective effect on the brain, thereby inhibiting the activity of pro-inflammatory cytotoxicity and inflammatory pathways and exhibiting an overall anti-inflammatory action^24^. Apigenin primarily exists in its glycosylated form in vegetables, fruits, and herbs. It exerts anticancer effects on cancer cells through multiple pathways and is effective against various diseases, such as diabetes, memory loss, Alzheimer’s disease, depression, and insomnia^25^. Chlorogenic acid (CGA) is a phenylacrylate polyphenol produced by the shikimic acid pathway during aerobic respiration. It is a compound obtained from plant extracts of various sources, including Lonicera japonica, potato, cork, chrysanthemum, strawberry, and mangoes. It has anti-inflammatory, antioxidant, antibacterial, antitumor, liver and kidney protective, glucose and lipid metabolism regulatory, and nervous system protective effects^26,27^. Myricetin belongs to the polyphenol family and is found in berries, grapes, tea, fruits, and medicinal plants. Several studies reported that myricetin has antioxidant and free-radical scavenging properties; making it useful for immune response, treatment of noise-induced hearing loss and hypertension, and as an analgesic, anti-allergic, and anti-inflammatory agent. In addition, myricetin induces apoptosis by inhibiting this cascade in cancer cells and has anticancer effects^28,29^. In addition, few studies have investigated the pharmacological effects of Hexadec-11-enoic acid and saponin II in the body. Further studies should be conducted with other fractions or constituents to identify the active compounds responsible for the effects of MS.

In the development of anticancer drugs, c-Myc is an essential target gene. In various cancers, including breast, colon, lung, and pancreatic cancers, c-Myc is overexpressed. Thus, c-Myc is a potent chemotherapeutic target^30,31^. A previous study reported that CNOT2 induces apoptosis in cancer cells by regulating c-Myc expression. It can be concluded that the MS extract induces apoptosis through this mechanism. Based on this information, we confirmed that the MS extract inhibited pancreatic cancer cell growth and proliferation using the MTT assay and colony formation (Fig. 1). Immunoblotting data confirmed that the MS extract induced the protein expression levels of the cleaved form of PARP and caspase 3, which are related to apoptosis in pancreatic cancer cells, in a concentration- and time-dependent manner. TUNEL assay and Annexin V/PI staining also confirmed that MS extract indued apoptotic cell death in PC (Fig. 3). In addition, we found that the MS extract reduced the protein expression level of c-Myc, the stability of the c-Myc protein, and its sensitivity to FBS (Figs. 4, 5). Among the results, Skp2, which regulates the ubiquitination of c-Myc, showed a decrease in protein expression due to MS treatment (Fig. 4E). Skp2, or S-phase kinase-associated protein 2, is a protein that plays an important role in regulating gene expression, cell division, and growth in the cell cycle. Previous studies have shown that Skp2 regulates the stability of c-Myc. Skp2 promotes the polyubiquitination of c-Myc, leading to its protein degradation. This weakens the stability of c-Myc and plays a role in suppressing cell cycle progression and cell growth by inhibiting the activation of genes regulated by c-Myc. However, Skp2 not only sends a signal for the destruction of c-Myc, but also acts as a strong activator of Myc transcriptional activity, ultimately functioning as an oncogene. So, when treated with MS that induce cancer cell death, the expression of Skp2 decreases simultaneously with c-Myc. In conclusion, Skp2 plays a role in coupling the destruction and activity of Myc through proteasomes, and Skp2-mediated activation of Myc appears to be an important process in controlling mammalian cell growth.We found that the oncogenic role of CNOT2 is supported by available bioinformatics databases: CNOT2 was overexpressed in tumor tissues than in normal tissues in patients with PC, and high expression rates of CNOT2 correlated with lower survival rates in patients with cancer. These results show that CNOT2 acts as a tumor-inducing factor in pancreatic cancer. CNOT2 and c-Myc were proportionally associated with pancreatic cancer. The MS extract inhibited c-Myc expression via CNOT2 (Fig. 6). Additionally, CNOT2 was proportionally related to migration-related factors in pancreatic cancer, and the MS extract inhibited migration through CNOT2 (Fig. 7).

## Conclusion

**Figure 9:**
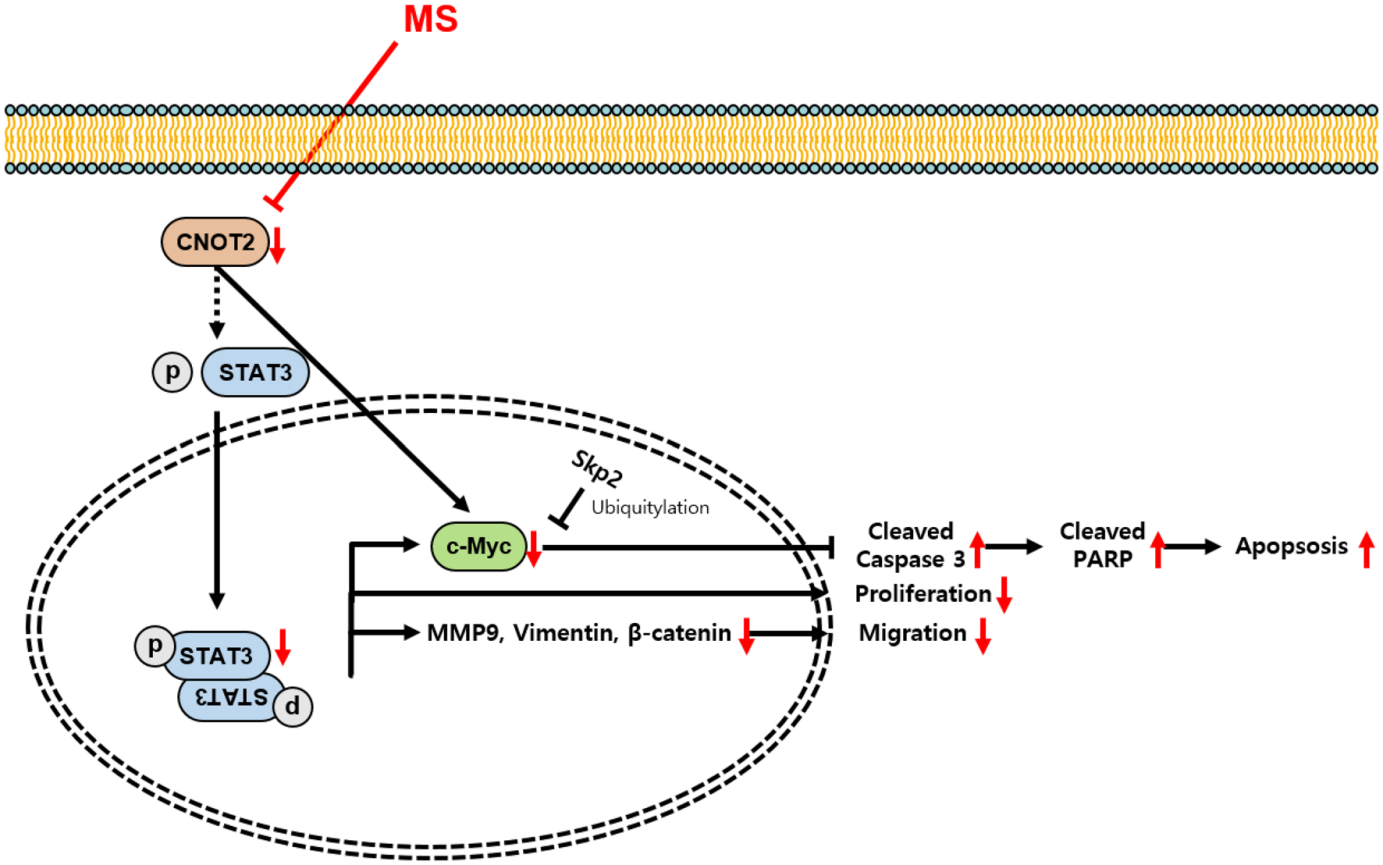
Overall scheme of MS extract working pathway in pancreatic cancer cells. The MS extract exhibits an anti-cancer effect in pancreatic cancer by regulating the oncogene c-Myc. This process involves the regulation of apoptotic and migration-related factors through CNOT2, leading to decreased stability of c-Myc and reduced sensitivity to nutrients. Additionally, the MS extract increases the sensitivity of pancreatic cancer cells to the existing chemotherapeutic drug, 5-FU, and shows promising results as a new anti-cancer agent.

In conclusion, our study clearly demonstrates that MS extract exhibits potent anti-cancer activity against pancreatic cancer cell lines (Fig. 9). The MS extract was found to increase the protein expression of cleaved caspase3 and cleaved PARP in both MIA PaCa-2 and Capan-2 cell lines. Moreover, it was revealed that MS induces apoptotic cell death through TUNEL assay and Annexin VI/PI staining. Additionally, it was confirmed that MS extract regulates the activity of Skp2, thereby inhibiting the expression and stability of c-Myc. Furthermore, it was observed that CNOT2, which primarily acts as an oncogene in various cancers, also functions as an oncogene in pancreatic cancer and is associated with migration. The study highlighted that MS extract suppresses c-Myc expression by regulating CNOT2 and inhibits migration by modulating STAT3 signaling. Moreover, when combined with the conventional anticancer drug 5-FU, a combinational effect was observed. These research findings suggest that MS has the potential to be developed as a novel anticancer agent. However, further elucidation and validation of these observations in vivo are required.

## Methods

### Reagents

Primary antibodies for c-Myc were purchased from Abcam (Cambridge, UK); PARP, caspase 3, CNOT2, p-STAT3 (T705), STAT3, histone H3, ribosomal protein L5, and Skp2 were purchased from Cell Signaling Technology (Beverly, MA, USA); and MMP9, β-actin, and secondary antibodies were purchased from Santa Cruz Biotechnology (Dallas, TX, USA).

### Cell culture

MIA PaCa-2 and Capan-2 cells were obtained from the Korea Cell Line Bank (KCLB, Seoul, Korea). The MIA PaCa-2 and Capan-2 cell lines were cultured in Dulbecco’s modified Eagle’s medium (DMEM) and Roswell Park Memorial Institute 1640 (RPMI-1640) (Corning Inc., New York, NY, USA), respectively. All cells were cultured in a medium containing 10% fetal bovine serum (Gibco, Grand Island, NY, USA) and 1x penicillin-streptomycin solution (Gibco BRL, Paisley, Scotland) at 37 °C and 5 % CO2 conditions.

### Preparation of momordicae Semen extract

The plant extract (CA02-029) used in this study was obtained from the Korea Plant Extract Bank of the Korea Research Institute of Bioscience and Biotechnology (Daejeon, Korea). A voucher specimen (PBC-118) was stored in the herbarium at the Korea Research Institute of Bioscience and Biotechnology. The plant (73 g) was dried in the shade and powdered, after which 1 L of95.0% ethyl alcohol (GR grade) was added, and the extract was obtained after completing 30 cycles (40 KHz, 1500 W, 15 min; and ultrasonication-120 min. per cycle) at room temperature using an ultrasonic extractor (SDN-900H; SD-ULTRASONIC Co., LTD, Seoul, Korea). After filtration (Qualitative Filter No.100, HYUNDAI MICRO Co., LTD, Seoul, Korea) and drying under reduced pressure, 4.3 g of the MS extract was obtained. The dried samples were dissolved in dimethyl sulfoxide for further experiments. All plant materials were deposited in the Plant Extract Bank of the Korea Research Institute of Bioscience and Biotechnology (KRIBB) in Daejeon, Korea (http://extract.kribb.re.kr/).

### MTT assay (Cell Viability Assay)

MIA PaCa-2 and Capan-2 cells were seeded at 1 × 10^4^ cells/well in 96-well plates and incubated overnight. Afterwards, to determine the cytotoxicity of MS, 50–250 μg/mL of MS extract was treated and incubated for 24 h. MTT (3-[4,5-dimethyl-2-thiazolyl]-2,5-diphenyl-[2H]-tetrazolium bromide) solution was added to a final concentration of 0.5 mg/mL. After incubation for 2 h, the medium was removed, and the formazan crystals that formed were dissolved in DMSO. The absorbance was measured at 540 nm using a microplate reader.

### Colony formation assay

MIA PaCa-2 and Capan-2 cells treated according to the experimental conditions were reseeded at 1 × 10^3^ cells/well in 6-well cell plates and incubated for 14 days. The colonies formed were stained using a Diff Quik Solution 2 kit (Sysmex Corporation, Kobe, Hyogo, Japan). The stained colonies were then dissolved in ethanol, and the absorbance was measured at a wavelength of 540 nm using a microplate reader.

### Immunoblotting

MIA PaCa-2 and Capan-2 cells were seeded in 6-well plates and incubated overnight. The cells were then treated according to the manufacturer’s instructions. Cell lysates were obtained using a cell lysis buffer (Cell Signaling Technology, Danvers, MA, USA), and cytoplasmic and nuclear fractions were obtained using NE-PER nuclear and cytoplasmic extraction reagents (Thermo Fisher Scientific, Waltham, MA, USA). Western blotting was performed as described by Jee et al.^32^. The membranes were incubated overnight at 4 °C with the following antibodies: c-Myc (1:1,000), PARP (1:1,000), Caspase 3 (1:1,000), Cleaved Caspase 3 (1:1,000), CNOT2 (1:1,000), L5 (1:1,000), Skp2 (1:1,000), MMP9 (1:1,000), p-STAT3(T705)(1:3,000), STAT3(1:1,000), Histone H3(1:2,000) and β-actin(1:10,000). Data were normalized to the housekeeping genes β-actin or histone H3 and quantified based on the control according to each condition.

### Immunofluorescence assay

MIA PaCa-2 cells were incubated at 37 °C with or without MS in a confocal dish for 24 h. Afterward, the cells were stained with c-Myc (1:500 diluted in PBS with 3% BSA), as described in previous studies. After the nuclei were stained with DAPI, the stained cells were observed using a CELENATM S Digital Imaging System (Logos Biosystems, Inc. Anyang-si, Gyeonggi-do, South Korea).

### TUNEL assay

The DeadEnd™ Fluorometric TUNEL system kit (Promega, Madison, WI, USA) was used to confirm the apoptotic effect of MS extracts on MIA PaCa-2 cells. Stained cells were visualized using the CELENA™ S Digital Imaging System (Logos Biosystems, Inc. Anyang-si, Gyeonggi-do, South Korea).

### Annexin V/PI staining

The cells were washed with PBS and resuspended in PBS containing Annexin V antibody tagged with FITC and PI staining solution for 30 minutes at room temperature. The nuclei were subsequently counterstained with DAPI, and apoptosis of the cells was analyzed using the CELENATM S Digital Imaging System.

### RT-qPCR

Cells were seeded at 30 × 10^4^ cells/well in 6-well plates and incubated with or without MS for 24 h. The cells were then treated with RiboEx to extract RNA using the GeneAll Hybrid-R RNA Purification Kit (GeneAll, Seoul, Korea). cDNA synthesis and qPCR were performed as previously described. AccuTarget™ Human Real-Time PCR Primers (100 reactions) were purchased from Bioneer (Daejeon, Korea) and normalized to GAPDH to determine mRNA levels.

### Transfection

MIA PaCa-2 cells were seeded at a density of 7 × 10^4^ cells/well in 6-well plates and incubated overnight. Using a transfection reagent (INTERFERin, Polyplus, France), L5 siRNA or Skp2 siRNA (Santa Cruz Biotechnology, Dallas, TX, USA), and CNOT2 siRNA or scrambled siRNA control (Bioneer, Daejeon, Korea) were transfected. The transfected cells were cultured for 48–72 h, depending on the experimental conditions.

### Protein stability Assay for c-Myc stability

Cells treated with DMSO or MS (250 μg/mL) for 24 h were treated with 50 μg/mL cycloheximide (CHX; Merck KGaA, Darmstadt, Germany) at the indicated time points, and western blotting was performed.

### Wound healing assay

When the cells were incubated to approximately 100 % confluence in 6-well plates, a 200 μL pipette tip was used to make a straight scratch. The wounded monolayers were gently washed with PBS (phosphate-buffered saline) and photographed under a microscope (0 h). After treating the cells with DMSO or MS for 24 h, the degree of cell migration was determined using a microscope.

### LC–MS

Chromatographic separation of the extract was performed using liquid LC-MS. The experiment was conducted in accordance with previous studies ^33,34^. For compound profiling, data were analyzed using MassHunter Qualitative Analysis software (version B 07.00, Agilent Technologies, Santa Clara, CA, USA).

### Analysis of *CNOT2* expression in patients with PC

We used the TIMER2.0 online tool (http://timer.cistrome.org/) to investigate *CNOT2* gene expression between tumors and adjacent normal tissues and the association between *CNOT2* expression and survival status in patients with PAAD. The data were analyzed using the Wilcoxon statistical test, and a *P*-value < 0.05 was considered statistically significant. Survival data were represented by the Kaplan–Meier curve of the gene. In addition, we investigated the association between the gene of interest, *CNOT2*, the oncogenic factor MYC, and EMT markers (VIM, MMP9, STAT3, and CTNNB1) in PAAD. Data were analyzed using Spearman’s correlation test, and statistical significance was set at P < 0.05 were considered significant.

### Statistical analysis

Comparisons between multiple groups were analyzed using ANOVA and Tukey’s test, and comparisons between 1:1 groups were performed using the unpaired *t*-test (one-tailed). Statistical significance was set at *P* < 0.05; *P* < 0.01, *P* < 0.001, and *P* < 0.0001 were considered highly significant. All data are presented as the mean ± SEM.

### Supplementary Information

Supplementary Figures.

## Data Availability

All data generated or analyzed in this study are included in the supplementary information files.
