# Supplementary material for: Momordicae Semen inhibits migration and induces apoptotic cell death by regulating c-Myc and CNOT2 in human pancreatic cancer cells
Source: Sci Rep. 2023 Aug 7;13:12800. doi: 10.1038/s41598-023-39840-w (PMC10406802; doi:10.1038/s41598-023-39840-w)
Supplement: Supplementary file 1 — Supplementary Figures. [file 41598_2023_39840_MOESM1_ESM.pdf]

Figure 3A

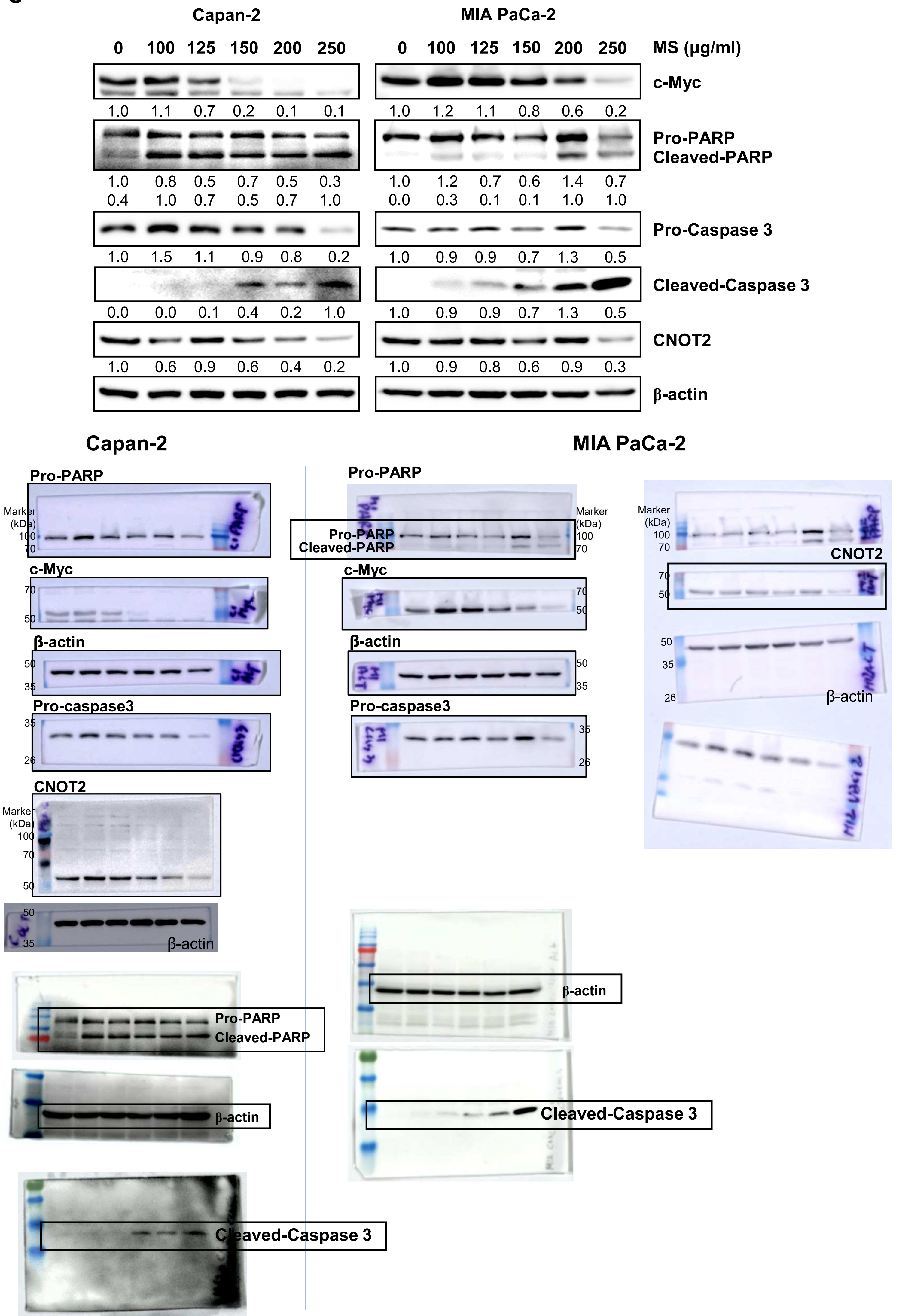

Capan-2
MIA PaCa-2
0 100 125 150 200 250
0 100 125 150 200 250
MS (μg/ml)
c-Myc
1.0 1.1 0.7 0.2 0.1 0.1
1.0 1.2 1.1 0.8 0.6 0.2
Pro-PARP
Cleaved-PARP
1.0 0.8 0.5 0.7 0.5 0.3
0.4 1.0 0.7 0.5 0.7 1.0
1.0 1.2 0.7 0.6 1.4 0.7
0.0 0.3 0.1 0.1 1.0 1.0
Pro-Caspase 3
1.0 1.5 1.1 0.9 0.8 0.2
1.0 0.9 0.9 0.7 1.3 0.5
Cleaved-Caspase 3
0.0 0.0 0.1 0.4 0.2 1.0
1.0 0.9 0.9 0.7 1.3 0.5
CNOT2
1.0 0.6 0.9 0.6 0.4 0.2
1.0 0.9 0.8 0.6 0.9 0.3
β-actin
Capan-2
MIA PaCa-2
Pro-PARP
Marker (kDa)
100
70
Pro-PARP
Cleaved-PARP
c-Myc
70
50
β-actin
50
35
Pro-caspase3
35
26
CNOT2
Marker (kDa)
100
70
50
β-actin
CNOT2
26
Pro-PARP
Cleaved-PARP
β-actin
Cleaved-Caspase 3

Cell cycle revision data

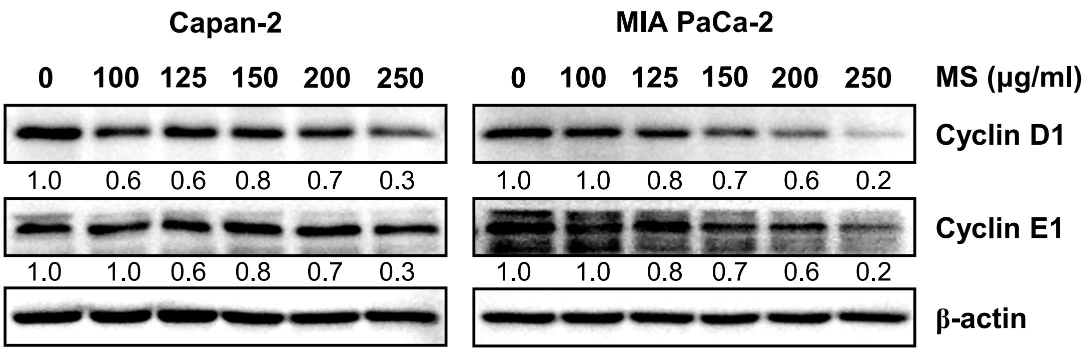

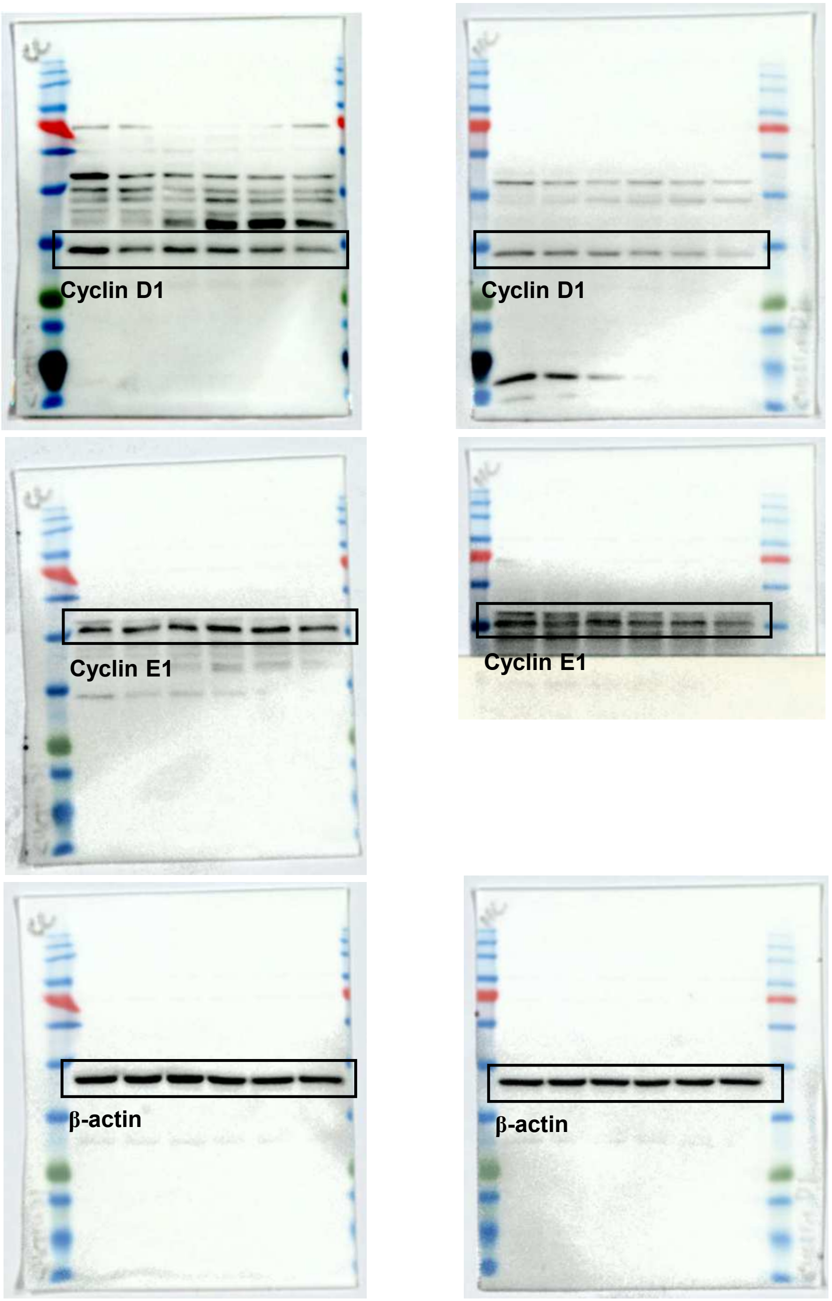

Figure 3B

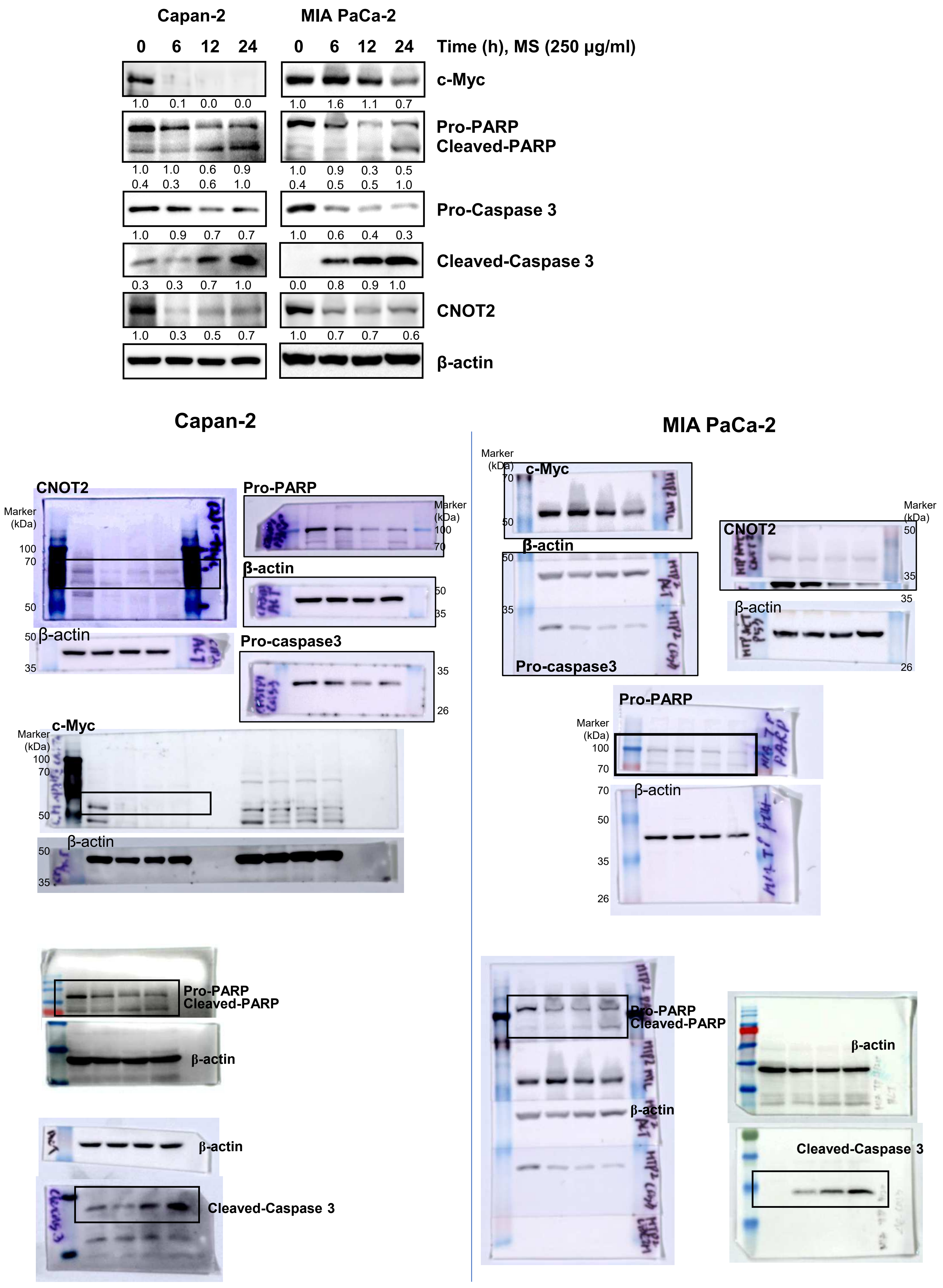

Capan-2
MIA PaCa-2
0 6 12 24
0 6 12 24
Time (h), MS (250 μg/ml)
c-Myc
1.0 0.1 0.0 0.0
1.0 1.6 1.1 0.7
Pro-PARP
Cleaved-PARP
1.0 1.0 0.6 0.9
0.4 0.3 0.6 1.0
1.0 0.9 0.3 0.5
0.4 0.5 0.5 1.0
Pro-Caspase 3
1.0 0.9 0.7 0.7
1.0 0.6 0.4 0.3
Cleaved-Caspase 3
0.3 0.3 0.7 1.0
0.0 0.8 0.9 1.0
CNOT2
1.0 0.3 0.5 0.7
1.0 0.7 0.7 0.6
β-actin
Capan-2
MIA PaCa-2
Marker (kDa)
CNOT2
Pro-PARP
β-actin
Pro-caspase3
c-Myc
Pro-PARP
Cleaved-PARP
β-actin
Cleaved-Caspase 3
100
70
50
35
26

Figure 4

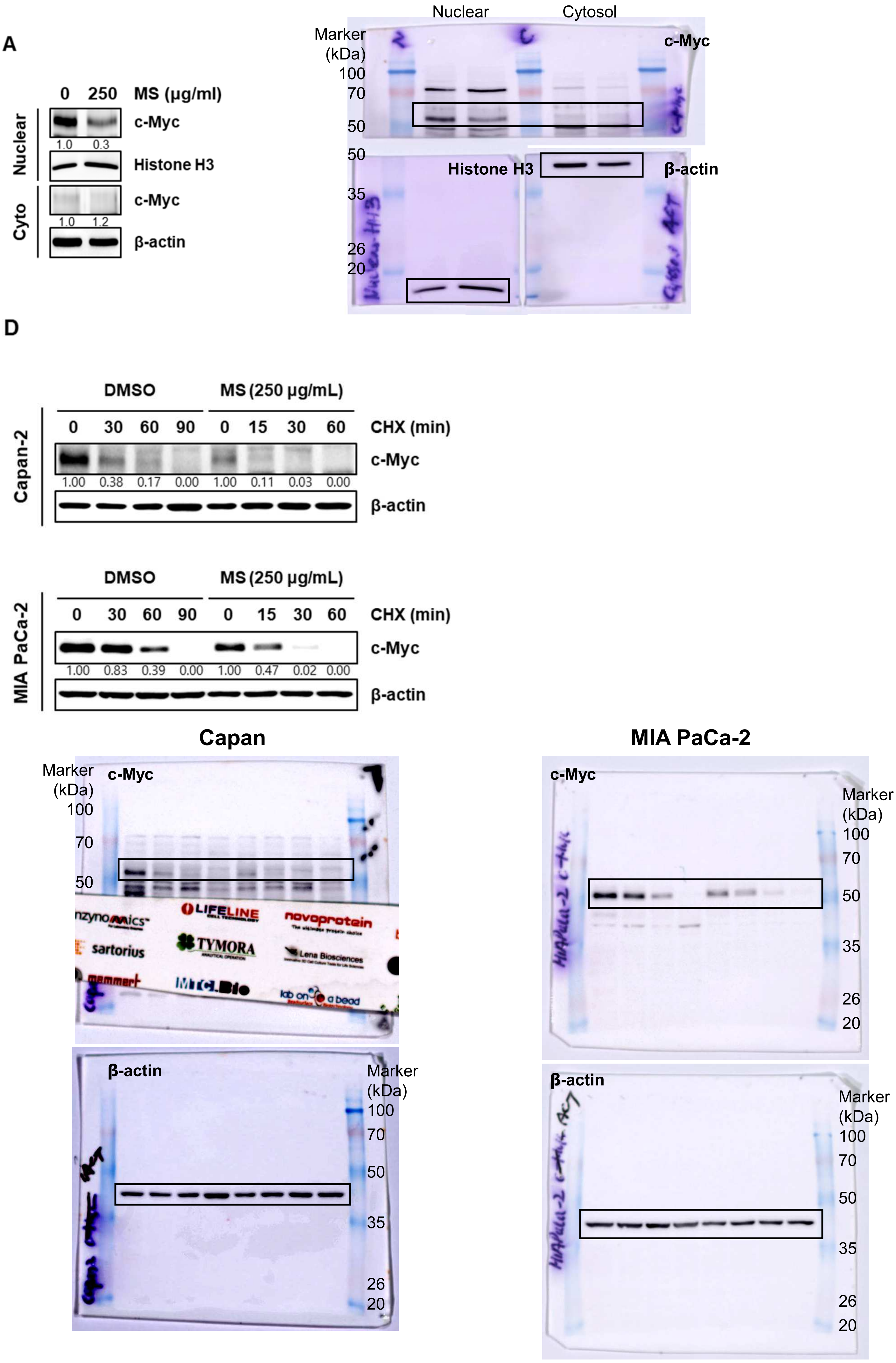

A
0
250
MS (μg/ml)
Nuclear
c-Myc
1.0
0.3
Histone H3
Cyto
c-Myc
1.0
1.2
β-actin
Nuclear
Cytosol
Marker
(kDa)
100
70
50
c-Myc
50
35
26
20
Histone H3
β-actin
D
DMSO
MS (250 μg/mL)
Capan-2
0
30
60
90
0
15
30
60
CHX (min)
c-Myc
1.00
0.38
0.17
0.00
1.00
0.11
0.03
0.00
β-actin
DMSO
MS (250 μg/mL)
MIA PaCa-2
0
30
60
90
0
15
30
60
CHX (min)
c-Myc
1.00
0.83
0.39
0.00
1.00
0.47
0.02
0.00
β-actin
Capan
MIA PaCa-2
Marker
(kDa)
100
70
50
c-Myc
LIFELINE
novoprotein
sartorius
TYMORA
Lena Biosciences
MTC.Bio
lab on a bead
c-Myc
Marker
(kDa)
100
70
50
35
26
20
β-actin
Marker
(kDa)
100
70
50
35
26
20
β-actin
Marker
(kDa)
100
70
50
35
26
20

Figure 4

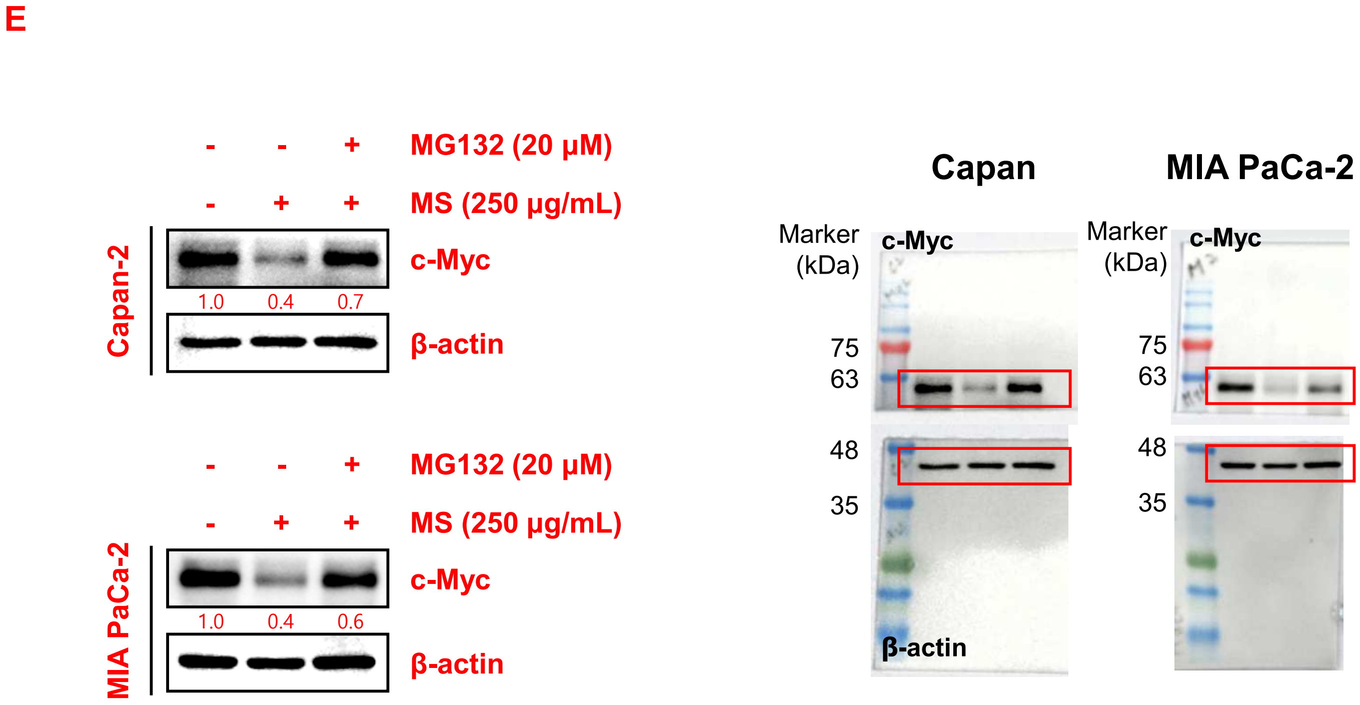
E
- - + MG132 (20 µM)
- + + MS (250 µg/mL)
Capan-2
c-Myc
1.0 0.4 0.7
β-actin
- - + MG132 (20 µM)
- + + MS (250 µg/mL)
MIA PaCa-2
c-Myc
1.0 0.4 0.6
β-actin
Capan
MIA PaCa-2
Marker (kDa)
c-Myc
75
63
48
35
β-actin

Figure 4

F

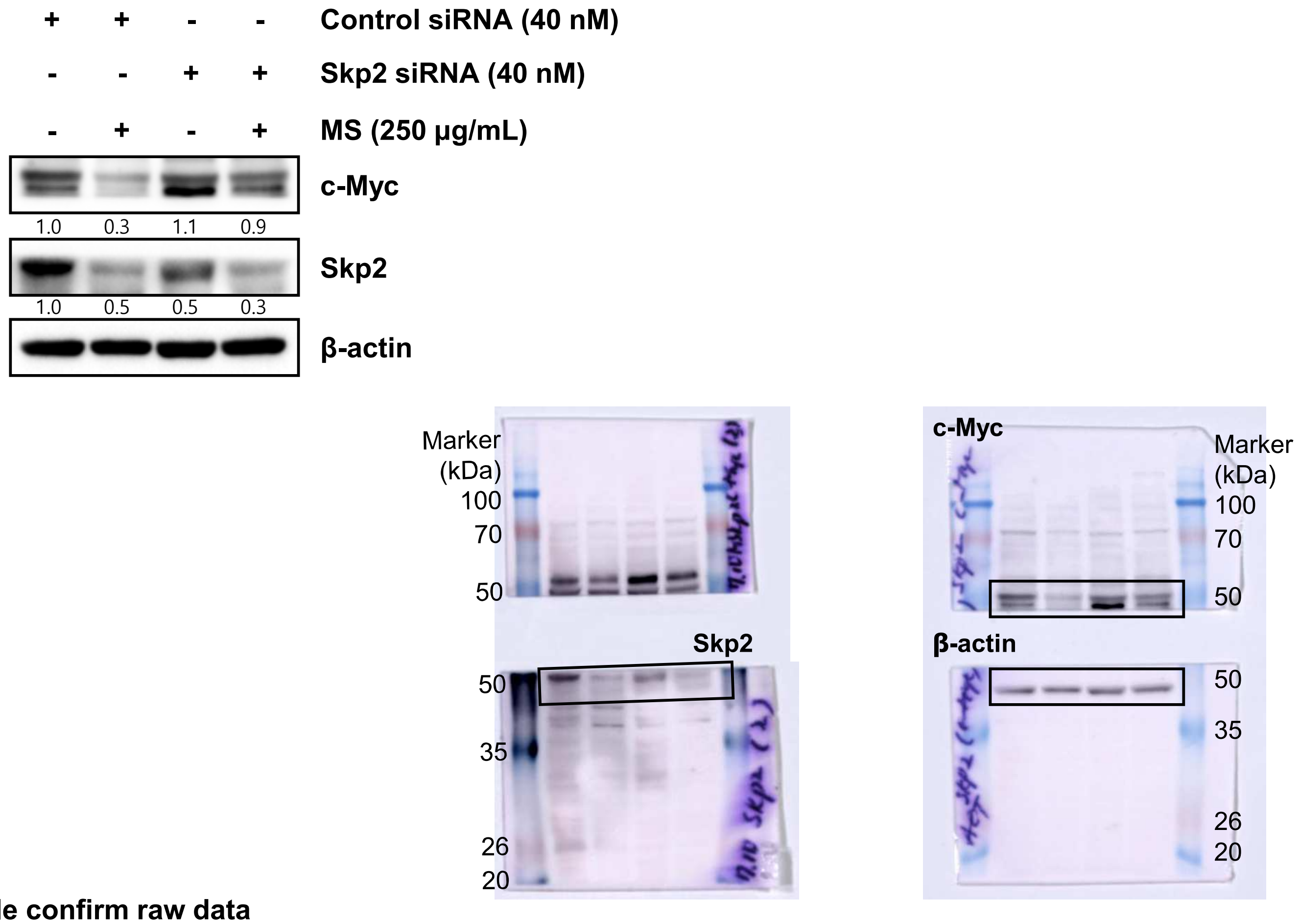

Double confirm raw data

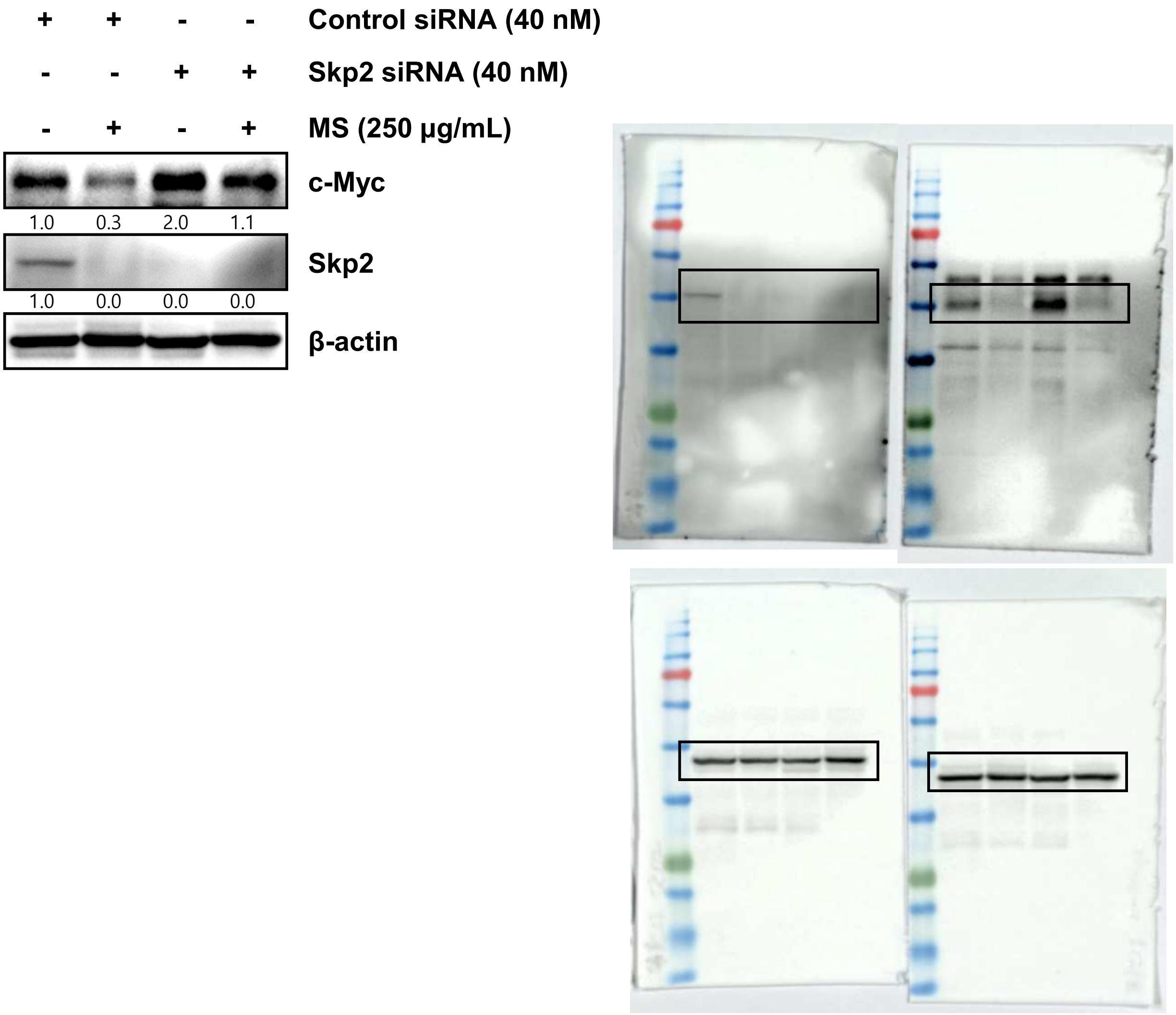

Figure 5

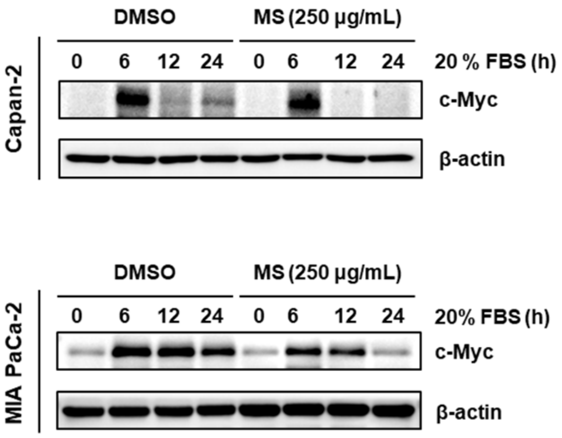

Capan-2

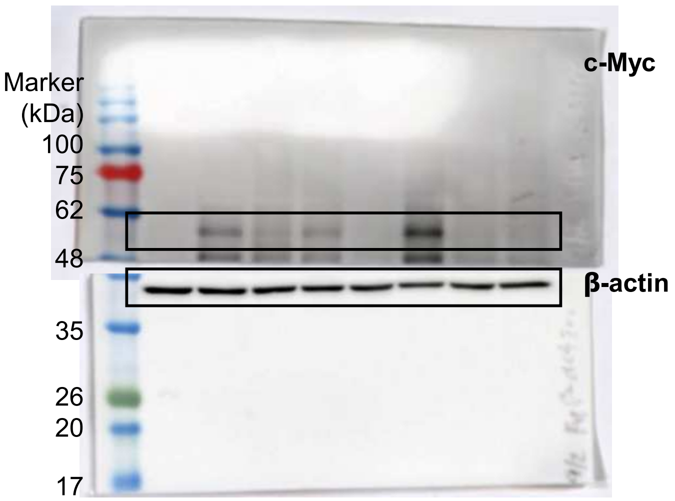

MIA PaCa-2

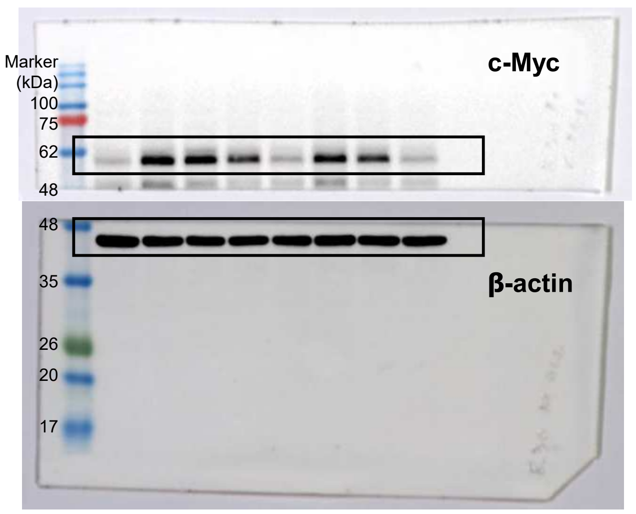

Figure 6

C

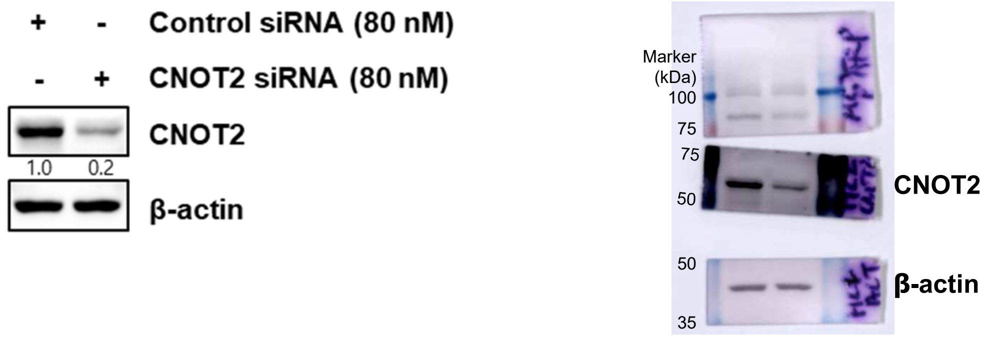

+ - Control siRNA (80 nM)
- + CNOT2 siRNA (80 nM)
CNOT2
1.0 0.2
β-actin
Marker (kDa)
100
75
75
50
50
35
CNOT2
β-actin

G

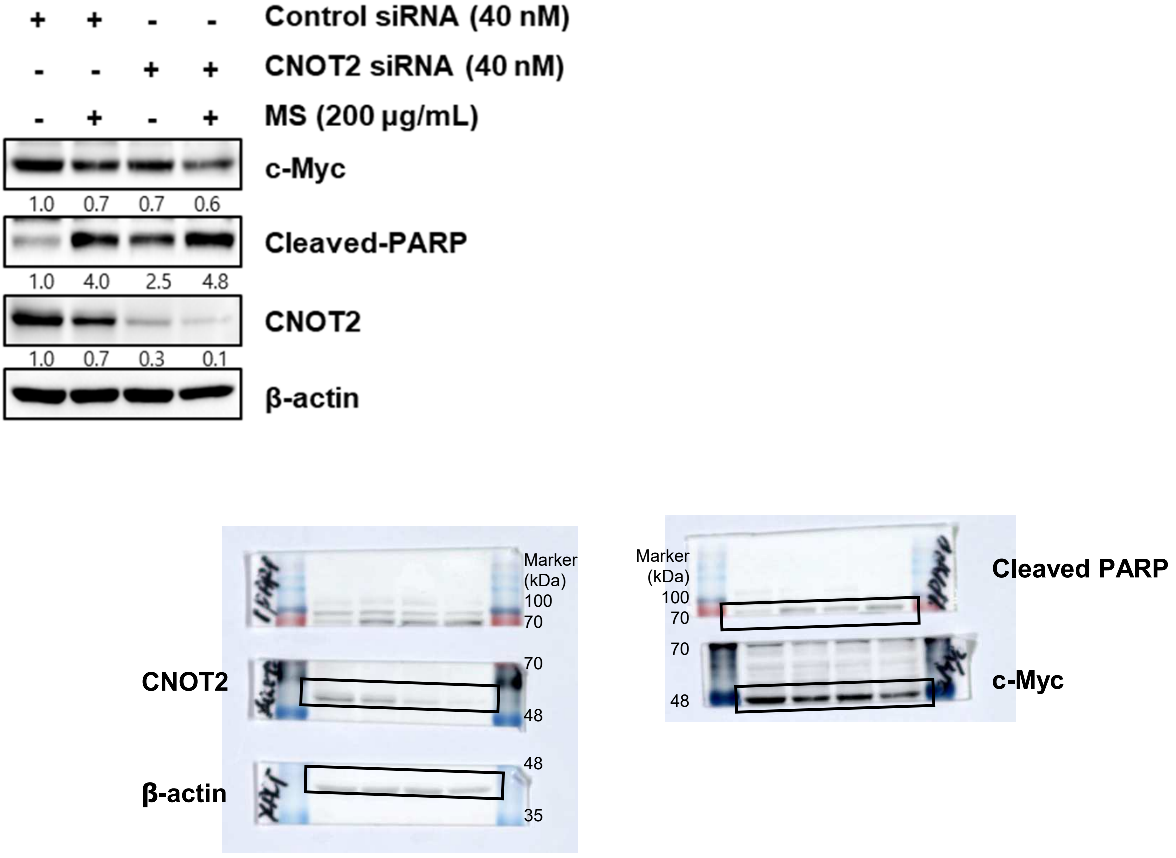

+ + - - Control siRNA (40 nM)
- - + + CNOT2 siRNA (40 nM)
- + - + MS (200 µg/mL)
c-Myc
1.0 0.7 0.7 0.6
Cleaved-PARP
1.0 4.0 2.5 4.8
CNOT2
1.0 0.7 0.3 0.1
β-actin
Marker (kDa)
100
70
70
48
48
35
CNOT2
β-actin
Marker (kDa)
100
70
70
48
Cleaved PARP
c-Myc

Figure 7

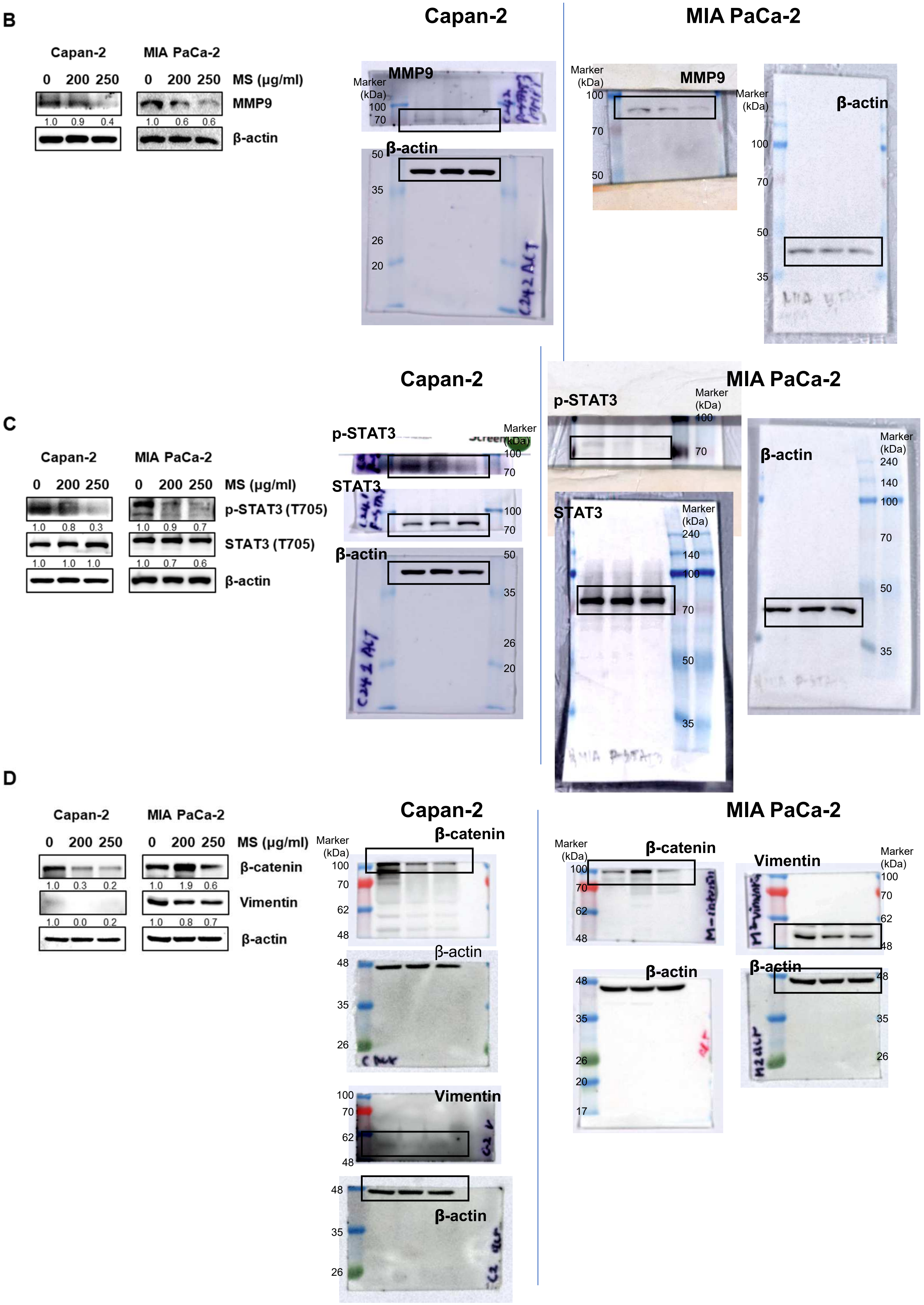

B
Capan-2
MIA PaCa-2
0 200 250 0 200 250 MS (μg/ml)
MMP9
1.0 0.9 0.4 1.0 0.6 0.6
β-actin
Capan-2
MIA PaCa-2
MMP9
Marker (kDa)
100
70
50
35
26
20
β-actin
C
Capan-2
MIA PaCa-2
0 200 250 0 200 250 MS (μg/ml)
p-STAT3 (T705)
1.0 0.8 0.3 1.0 0.9 0.7
STAT3 (T705)
1.0 1.0 1.0 1.0 0.7 0.6
β-actin
p-STAT3
STAT3
240
140
D
Capan-2
MIA PaCa-2
0 200 250 0 200 250 MS (μg/ml)
β-catenin
1.0 0.3 0.2 1.0 1.9 0.6
Vimentin
1.0 0.0 0.2 1.0 0.8 0.7
β-actin
62
48
17

Figure 8

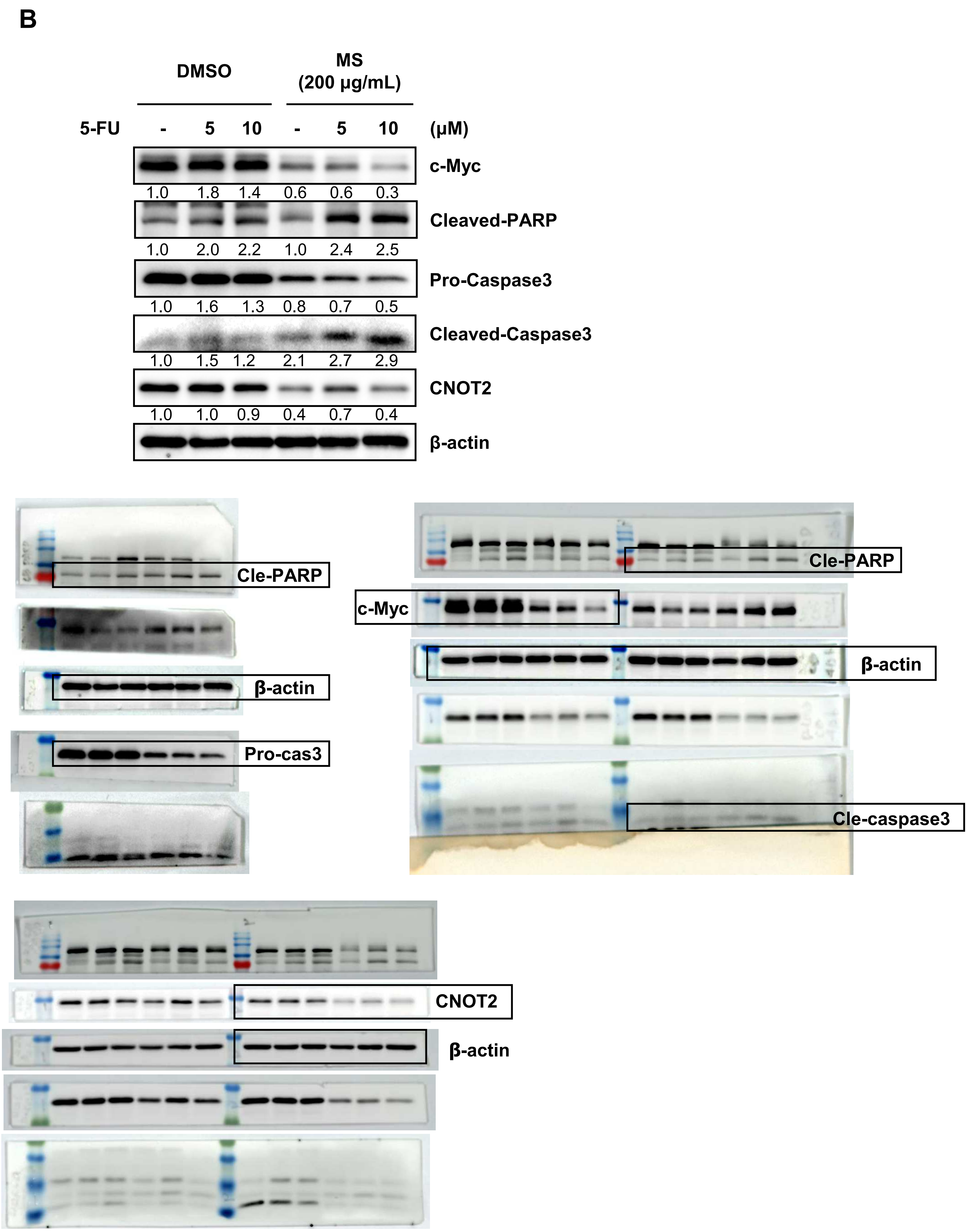
B
DMSO
MS
(200 μg/mL)
5-FU
-
5
10
-
5
10
(μM)
c-Myc
1.0 1.8 1.4 0.6 0.6 0.3
Cleaved-PARP
1.0 2.0 2.2 1.0 2.4 2.5
Pro-Caspase3
1.0 1.6 1.3 0.8 0.7 0.5
Cleaved-Caspase3
1.0 1.5 1.2 2.1 2.7 2.9
CNOT2
1.0 1.0 0.9 0.4 0.7 0.4
β-actin
Cle-PARP
β-actin
Pro-cas3
Cle-PARP
c-Myc
β-actin
Cle-caspase3
CNOT2
β-actin
